# A multi-country citizen-science study on what makes us enjoy a cup of coffee

**DOI:** 10.1038/s41538-026-00832-5

**Published:** 2026-04-22

**Authors:** Georgiana Juravle, Delia Elena Diaconașu, Ana-Maria Andrei, Felipe Reinoso-Carvalho, Simon D’Alfonso, Valentina Goglio, Kosuke Motoki, Simone Schmidt, Ceyhun Uçuk, George Van Doorn, Fabiana Mesquita Carvalho, Charles Spence

**Affiliations:** 1 Sensorimotor Dynamics Laboratory, Faculty of Psychology and Education Sciences, Alexandru Ioan Cuza University, Iasi, Romania; 2 Department of Social Sciences and Humanities, Institute of Interdisciplinary Research, Alexandru Ioan Cuza University, Iasi, Romania; 3 Faculty of Psychology and Educational Sciences, Stefan cel Mare University, Suceava, Romania; 4 School of Management, Universidad de los Andes, Bogotá, Colombia; 5 School of Computing and Information Systems, University of Melbourne, Parkville, VIC Australia; 6 Department of Cultures, Politics and Society, University of Turin, Turin, Italy; 7 Faculty of Business and Commerce, Keio University, Tokyo, Japan; 8 Gastronomy and Culinary Arts Department, Tourism Faculty, Gaziantep University, Gaziantep, Turkey; 9 Gastronomy and Culinary Arts Department, Faculty of Applied Science, Istanbul Gelisim University, Istanbul, Turkey; 10 Institute of Health and Wellbeing, Gippsland Campus, Federation University Australia, Churchill, VIC Australia; 11 Health Innovation and Transformation Centre, Mt Helen Campus, Federation University Australia, Ballarat, VIC Australia; 12 Department of Food Science and Nutrition, Faculty of Food Engineering, State University of Campinas, Campinas, Brazil; 13 Crossmodal Research Laboratory, Department of Experimental Psychology, Oxford University, Oxford, UK

**Keywords:** Psychology, Psychology, Science, technology and society

## Abstract

Many coffee preferences are known, but studies that comprehensively integrate *simultaneous* contributions to coffee enjoyment are lacking. An online citizen-science questionnaire designed to identify those factors associated with *momentary* coffee liking, surveying demographics, extrinsic/intrinsic qualities, and coffee-related habits, is presented (*N* = 2987; 5 continents; 7 languages; 11 countries; 77 nationalities). The results indicate a higher liking for coffee consumed black, during spring, in the morning, on Wednesdays, and from ceramic cups. Higher-priced coffee is appreciated significantly more, and liking-consumption quantity appears best-optimised at 4–5 cups/day. Several key characteristics for coffee-dislike are evident: waking-up late, drinking from a cup with a lid on, at noon during autumn, with cream, and, potentially implying a possible coping mechanism for bitterness-disliking, adding sugar to coffee. These results constitute the first multi-country cross-context integration of momentary coffee liking and provide an empirical foundation for context-sensitive models linking sensory/behavioural/temporal factors in beverage preference research.

After water and tea, coffee is the world’s third most consumed beverage and the second most traded commodity^1^, with significant cultural, economic, and social importance. Coffee has recently experienced a global rise in popularity and secured its status as an icon of the world marketplace^2^. Global consumption continues to expand and diversify across demographic groups and cultural contexts, increasing from 104.6 million 60-kg bags in 2000 to 173 million 60-kg bags in 2023^3^. Coffee production tends to be concentrated in low- and middle-income countries (i.e., about 70% of supply originates from Brazil, Vietnam, Colombia, and Indonesia) whereas demand is concentrated in middle- and high-income markets, with the United States (US), the European Union (EU), Brazil, and Japan together accounting for more than two-thirds of global consumption.

Health-wise, moderate coffee consumption (e.g., 3–5 cups/day) is associated with favourable health outcomes, including a lower risk of cardiovascular disease, type 2 diabetes, liver disease, obesity, metabolic syndrome, several cancers, as well as lower all-cause mortality^4,5^; though recommendations for moderate/low coffee consumption are made for diverse underlying medical health conditions and prescribed medicines. Given coffee’s widespread consumption, depending on the specific outcome considered, even modest relative risk reductions could yield meaningful public health benefits^5^. Emerging work also suggests that the timing of coffee intake may modify these associations. For instance, Wang and her colleagues^6^ reported that, relative to non-consumption, both moderate and heavy coffee intake were associated with lower all-cause mortality among morning-type drinkers, but no significant association was observed among all-day-type drinkers.

Moreover, coffee consumption closely aligns with contemporary lifestyles, with diverse factors considered to contribute to people’s enjoyment of coffee, including consumer demographics (age, sex), sensory attributes (flavour profiles, various additions to the coffee, such as sugar or milk), and a selection of basic consumption contexts and individual consumption preferences and/or habits (e.g., the time of day at which coffee is consumed). Take, for example, the stratification in Loftfield et al.’s study^7^, indicating that men and older adults (≥30 years) consume more daily coffee, while female consumers tend to spend more on higher-priced (i.e., also referred to as speciality) coffee types^8^. Further, cross-cultural comparisons point to sensory (taste, flavour) and functional (stimulant) motives as dominant determinants of coffee consumption, broadly similar across countries and cultures (see ref. ^9^, for a review). By contrast, specific habits and preferences linked to occasions, locations, and consumption contexts tend to vary with traditions and cultural norms. However, studies are presently lacking that comprehensively integrate the diverse contextual factors that may *simultaneously* contribute to the enjoyment of coffee. For instance, while diurnal patterns of consumption are known, how these interact with seasonal changes or specific added ingredients to coffee remains less clear^10,11^. Furthermore, from a consumer’s point of view, the influence of generational cohorts on coffee appreciation in the current market landscape is an evolving area that may be considered worthy of further study. A recent industry report highlights that coffee habits differ markedly by generation: Younger cohorts report drinking less coffee, they express a stronger orientation toward *third-wave* or specialty coffee, as well as expressing a preference for milk-forward coffee beverages, whereas Generation X and Baby Boomers more often nominate espresso as their primary choice^12^. In a similar manner, another recent study links younger consumers’ coffee choices to identity expression and social-media-mediated café experiences, thus marking a clear departure from the habit- and tradition-driven approach of older generations^13^. Notably, the social-media-sharing of coffee-to-go consumption appears substantially higher among younger cohorts than amongst Baby Boomers and older adults; though the social media sharing seems to be a food-related phenomenon nowadays, not only restricted to coffee^14^. Relatedly, a consumer’s nationality and/or origin with respect to coffee-(non-)producing regions represents an under-explored area at present.

Factors such as the impact of the coffee’s extrinsic qualities, including the receptacle (e.g., ceramic vs. disposable cups) and the actual price, on the drinker’s subjective liking of the coffee within needs consideration. Visual properties of the cup—such as its colour or material qualities—have been shown to influence sensory and hedonic judgements of specialty coffee^15,16^, though they may exert less of an influence on instant coffee^17^. Price and the coffee’s perceived quality appear to be linked in the mind of the consumer, with price being an important, premium-bearing attribute of coffee described in the literature^8^. At the market level, mainstream coffee demand is relatively price-inelastic. That is, consumers typically maintain their coffee consumption habits, despite the future coffee markets’ price volatility^18^. By contrast, among younger consumers—particularly in specialty/third-wave contexts—coffee is often perceived as an expensive commodity, a conspicuous-consumption signal, pointing to greater price sensitivity and cohort-specific dynamics^13^. Finally, when considering the marketing price–quality effect, it is expected that stating a higher price should raise expectations of quality and liking. Indeed, consistent with the price–quality heuristic, higher prices raised consumers’ expectations before the tasting, but did not reliably increase the experienced quality or liking once the product was sampled^19^. Understanding these multifaceted influences is important for the coffee industry and the research community, from cafes personalising their offerings and ambiance^20^, through to marketers targeting specific demographics, and product developers interested in optimising the consumer’s coffee drinking experience.

The present study thus aimed to comprehensively investigate those presumably multi-dimensional factors impacting the enjoyment of a cup of coffee. For this, a multi-country citizen-science survey was conducted (i.e., a collaborative research project where numerous and diverse individuals—from typically different groups/countries—participate in collecting and/or analysing data, so as to answer specific scientific questions, and as such, to contribute to a shared body of knowledge with broad geographic/temporal distribution and scope; see ref. ^21^, for an example). Several key variables relating to coffee consumption, including coffee extrinsic characteristics (e.g., the coffee price, type of cup, coffee with a lid on, with/without company), the presence of common additions to one’s coffee (e.g., sugar, milk, cream), consumer characteristics (e.g., generation), temporal factors (i.e., season, weekday, time of the day), as well as typical habits in relation to the consumed coffee (i.e., consumption frequency, waking hour) were recorded. To answer the study main question of what makes us enjoy a cup of coffee, the present study is structured around five central research aims: (1) The first aim is to investigate to what extent the extrinsic and/or intrinsic coffee attributes and consumption habits are associated with liking of the coffee, across different countries. (2) The second aim is to investigate how the interaction between specific coffee characteristics and temporal context relates to the present/momentary coffee enjoyment. (3) Third, we address if coffee characteristics are moderated by consumer demographics. (4) Fourth, we explore specificity in consumer evaluation, by researching how the perfect liking of a coffee (i.e., as evident in maximum ratings awarded to a consumed coffee), as opposed to average ratings liking, associate with distinct coffee characteristics of interest. And finally, (5) Given the nature of this study’s recruitment, we verify to what extent the observed associations are consistent across the sampled geographical locations, and whether robustness of our findings is maintained when accounting for geographical imbalances and recruitment-related dependencies. The present work is important and timely because it emphasises that the perfect cup of coffee is a dynamic expression, influenced by psychology, culture, and context, including geographical location of consumption, as much as by the coffee itself.

## Results

**Fig. 1:**
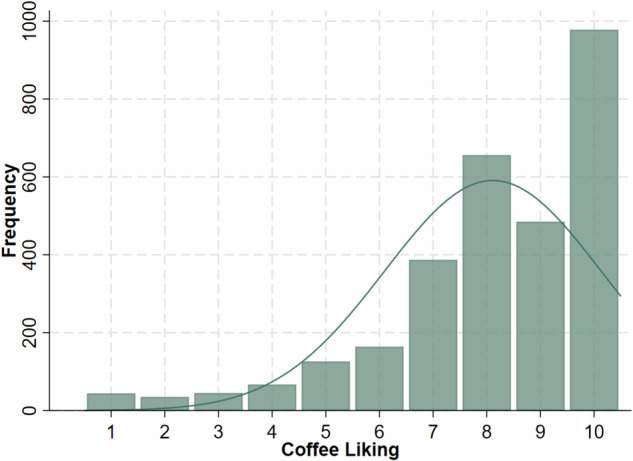
Histogram of the coffee liking (counts). *N* = 2987.

**Fig. 2:**
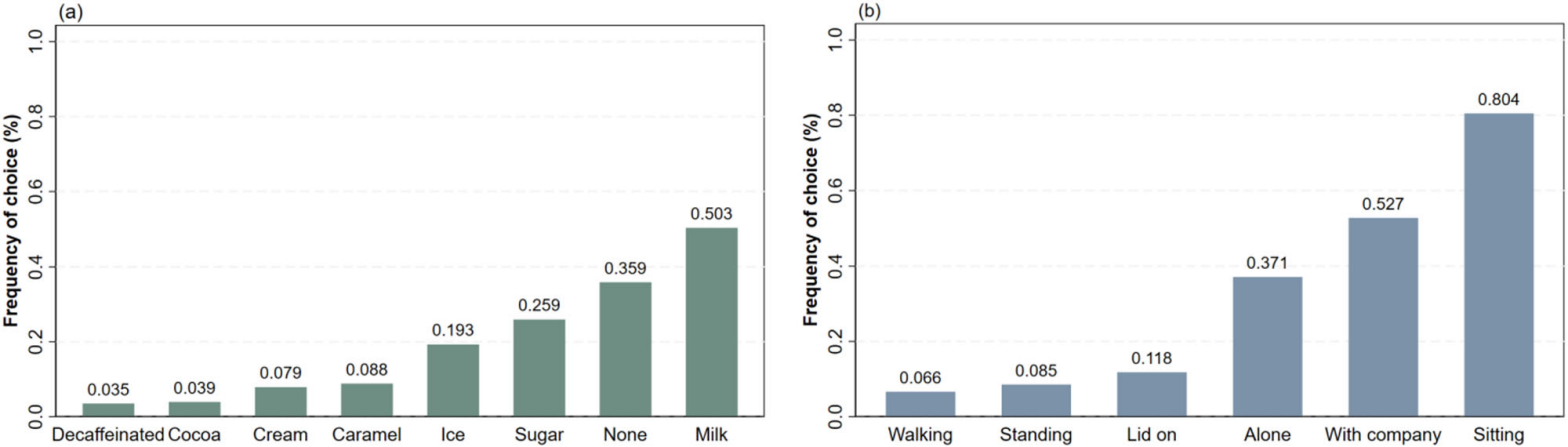
Reported frequency of choice of coffee qualities for the momentary coffee cup consumed by participants while they were filling in the survey, *N* = 2987. **a** Intrinsic coffee qualities and **b** Extrinsic coffee qualities.

**Fig. 3:**
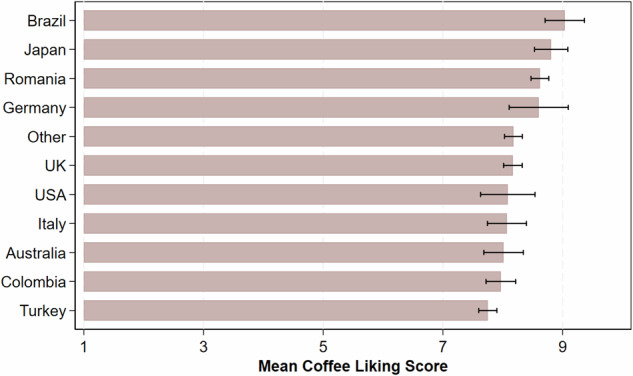
Coffee liking scores, means  ± 95% CIs, split according to the nationality reported by the participants. Nationals of countries accounting for less than 1% of the sample are aggregated under the category “Other” (67 countries, *N* = 344). Those participants who reported more than one nationality (*N* = 34), were considered in analysis with their first reported nationality.

Table 1 presents the survey summary statistics after excluding price outliers, *N* = 2987; see Table S1 for the distribution summary of recruitment country by month. Table 2 provides descriptive statistics for all continuous variables considered in the study. Figure 1 presents the distribution of the coffee Liking variable. Figure S1 in Supplementary Information presents the PPP-adjusted price distribution for the entire sample, *N* = 3002. Those reported intrinsic and extrinsic coffee qualities are depicted in Fig. 2. Figure 3 presents coffee preferences across different nationalities reported by the participants in the sample. Finally, additional graphical depictions of coffee Liking scores split according to different demographic, contextual, and product characteristics are presented in Fig. S2 of Supplementary Information.

**Table 1:** Survey summary statistics, counts (percentage)

	Study sample
*Language of the completed questionnaire*	*N* = 2987
English, *n* (%)	1006 (33.68)
Spanish, *n* (%)	269 (9.01)
Italian, *n* (%)	37 (1.24)
Japanese, *n* (%)	135 (4.52)
Portuguese, *n* (%)	76 (2.54)
Romanian, *n* (%)	411 (13.76)
Turkish, *n* (%)	1053 (35.25)
*Generation*^*a*^	*N* = 2983
Boomers, *n* (%)	113 (3.79)
Generation X, *n* (%)	416 (13.95)
Millennials/Generation Y, *n* (%)	1102 (36.94)
Generation Z, *n* (%)	1352 (45.32)
*Gender*	*N* = 2987
Female, *n* (%)	1761 (58.96)
Male, *n* (%)	1204 (40.31)
Other, *n* (%)	22 (0.74)
*Country development*^*b*^	*N* = 2979
Emerging and developing economies, *n* (%)	1965 (65.96)
Advanced economies, *n* (%)	1014 (34.04)
*Coffee-producing country*^*c*^	*N* = 2979
Producer, *n* (%)	597 (20.04)
Non-producer, *n* (%)	2,382 (79.96)
*Type of consumer*	*N* = 2987
Professional barista/roaster, *n* (%)	244 (8.17)
Regular consumer, *n* (%)	2743 (91.83)
*Season*^*d*^	*N* = 2987
Spring, *n* (%)	141 (4.72)
Summer, *n* (%)	1460 (48.88)
Autumn, *n* (%)	988 (33.08)
Winter, *n* (%)	398 (13.32)
*Weekday*^*d*^	*N* = 2987
Workweek, *n* (%)	2161 (72.35)
Weekend, *n* (%)	826 (27.65)
*Time of day*^*d*^	*N* = 2987
Morning, *n* (%)	1192 (39.91)
Noon/Afternoon, *n* (%)	1355 (45.36)
Evening, *n* (%)	440 (14.73)
*Type of cup*^*d*^	*N* = 2987
Plastic, *n* (%)	281 (9.41)
Paper, *n* (%)	406 (13.59)
Metallic, *n* (%)	35 (1.17)
Ceramic, *n* (%)	1689 (56.55)
Glass, *n* (%)	576 (19.28)
*Number of coffees consumed per day*^*e*^	*N* = 2987
1–2 cups, *n* (%)	1805 (60.43)
3–5 cups, *n* (%)	1051 (35.18)
More than 5 cups, *n* (%)	131 (4.39)
*Hours of sleep*^*f*^	*N* = 2987
Less than recommended, *n* (%)	682 (22.83)
As recommended, *n* (%)	1883 (63.04)
More than recommended, *n* (%)	422 (14.13)
*Waking hour*^*g*^	*N* = 2925
Before typical average waking time, *n* (%)	451 (15.42)
Average waking time, *n* (%)	2125 (72.65)
After typical average waking time, *n* (%)	349 (11.93)

^a^As discretised from standard generational split usage: Boomers born 1946 – 1964; Generation X born 1965 – 1980; Generation Y or Millennials born 1981 – 1996; Generation Z born 1996 – 2012. See https://www.pewresearch.org/short-reads/2019/01/17/where-millennials-end-and-generation-z-begins/, https://en.wikipedia.org/wiki/Generation.

^b^As derived from participants’ reported nationality. Variable discretised according to World economic outlook 2024 - International Monetary Fund, https://www.imf.org/en/Publications/WEO/Issues/2024/10/22/world-economic-outlook-october-2024.

^b^As derived from participants’ reported nationality. Producer countries: Bolivia, Brazil, China, Colombia, Cuba, Dominican Rep., Ecuador, El Salvador, Ethiopia, Guatemala, India, Indonesia, Kenya, Malaysia, Mexico, Peru, Philippines, Thailand, USA, Venezuela, and smaller scale producers, including Bangladesh, Ghana, Grenada, New Zeeland, Nigeria, Portugal, Saudi Arabia, Singapore, South Africa, Spain, and Taiwan. Variable discretised according to Foreign Agricultural Service, https://www.fas.usda.gov/data/production/commodity/0711100.

^c^As derived from participants’ responses with respect to the coffee they were drinking at the time of filling in the questionnaire.

^d^According to European Food Safety Authority—https://www.efsa.europa.eu/en/topics/topic/caffeine and U.S. Food and Drug Administration—https://www.fda.gov/consumers/consumer-updates/spilling-beans-how-much-caffeine-too-much—i.e., up to 400 mg of caffeine per day for healthy adults (about 3-5 coffee cups).

^e^According to American Academy of Sleep Medicine, based on a study on participants aged 18-60 years old^74^ and participants aged 12-17 years old: https://www.sleepfoundation.org/how-sleep-works/how-much-sleep-do-we-really-need.

^f^According to Walch et al.^75^, the average morning waking time is approximately 7 a.m. Waking patterns nevertheless vary by age group, with older individuals (i.e., older than 55 years of age) typically waking up between 6–8 a.m., while younger individuals ( < 30 years old) wake up between 7–9 a.m. Therefore, here, the categorial split of Wake-up hour was considered in relation to age. Wake-up time was discretized into three categories based on age-specific hour intervals: early risers included individuals aged ≤30 years who woke up between 4:00–6:00 a.m. and those over 30 years of age who woke between 4:00–5:00 a.m.; normal rise time referred to individuals aged ≤30 years with wake-up times between 7:00–9:00 a.m. and those over 30 years between 6:00–8:00 a.m.; and late risers comprised individuals aged ≤30 years waking between 10:00–11:00 a.m and those over 30 years of age between 9:00–11:00 a.m.

**Table 2:** Descriptive statistics

	Mean	SD	Min	Max	Skewness	Kurtosis	N
*Coffee Liking (1–10 VAS)*	8.11	2.02	1	10	−1.37	4.85	2987
*Coffee Price (international USD)*^*a*^	6.37	3.11	0.0006	28.43	1.22	6.76	2522
*Added ingredients (count)*	1.20	1.28	0	6	1.24	4.40	2987
*Number of coffees per day (count)*	2.50	1.52	1	10	1.70	7.33	2987
*Hours of sleep (count)*	7.68	1.84	4	12	0.92	3.68	2987
*Waking hour (h)*	7.41	1.47	4	11	0.32	2.85	2925
*Age (years)*	31.95	11.94	12	83	1.05	3.60	2983

^a^Coffee price was adjusted using World Bank^76^ PPP conversion factors for private consumption (LCU per international $), corresponding to the country where the coffee was consumed and the economic context occurred. The most recent available data were used for each country (i.e., 2024, though note that 2021 was used for Argentina, as more recent figures were unavailable).

For the group-based exploratory results, the analysis of variance (ANOVA) revealed a main effect of Generation on coffee preference (*F*[3, 468.29] = 6.64, *p* < 0.001, ${\eta }_{p}^{2}=0.007$), with Gen Z’s preference for coffee (*M* = 7.94, SD = 2.13) significantly lower compared to Gen X (*M* = 8.34, SD = 1.87; *t*(774.7) = 3.69, *p* = 0.001, *r* = 0.13) and Millennials (*M* = 8.24, SD = 1.89; *t*(2434.9) = 3.71, *p* = 0.001, *r* = 0.08). Second, a main effect of Season was observed on coffee preference (*F*[3, 600.73] = 14.18, *p* < 0.001, ${\eta }_{p}^{2}=0.013$). Coffee was liked best in spring (*M* = 8.55, SD = 1.40), significantly more than summer (*M* = 8.20, SD = 1.99; *t*(199.4) = 2.71, *p* = 0.037, *r* = 0.19) and autumn (*M* = 7.82, SD = 2.22; *t*(254.6) = 5.33, *p* < 0.001, *r* = 0.32). Further, coffee was liked least in the autumn, significantly less than summer (*t*(1958.5) = 4.37, *p* < 0.001, *r* = 0.10) and winter (*M* = 8.39, SD = 2.66; *t*(973.8) = −5.25, *p* < 0.001, *r* = 0.17). Third, a significant main effect of Weekday was also observed on the coffee Liking data (*F*[6, 1299.82] = 3.27, *p* = 0.003, ${\eta }_{p}^{2}=0.007$). Tuesday was the least preferred day to drink coffee (*M* = 7.84, SD = 2.22), with coffee Liking significantly lower than top-score-Wednesday (*M* = 8.35, SD = 1.90; *t*(825.5) = −3.605, *p* = 0.006, *r* = 0.12) and Sunday (*M* = 8.25, SD = 1.86; *t*(962.5) = −3.10, *p* < .0.033, *r* = 0.10). Fourth, the Time of day demonstrated a clear influence on coffee preference (*F*[2, 1135.60] = 21.45, *p* < 0.001, ${\eta }_{p}^{2}=0.015$). Morning coffee consumption was most liked most (*M* = 8.38, SD = 1.72), significantly better as compared to noon (*M* = 8.01, SD = 2.07; *t*(2536.4) = 4.96, *p* < 0.001, *r* = 0.10) and evening (*M* = 7.69, SD = 2.46; *t*(604) = 5.42, *p* < 0.001, *r* = 0.22). Noon consumption was liked significantly more, as compared to evening (*t*(653) = 2.45, *p* = 0.039, *r* = 0.10), with the latter showing the lowest preference.

Fifth, the Type of coffee cup significantly influenced coffee preference (*F*[4, 227.26] = 5.05, *p* < 0.001, ${\eta }_{p}^{2}=0.007$). Ceramic cups were liked best (*M* = 8.25, SD = 1.89), significantly surpassing plastic (*M* = 7.85, SD = 2.12; *t*(357.9) = −2.99, *p* = 0.024, *r* = 0.16) and paper cups (*M* = 7.82, SD = 2.22; *t*(553.9) = −3.76, *p* = 0.003, *r* = 0.16), which had the lowest liking scores. Sixth, a significant main effect was noted with respect to the addition of ingredients to the consumed coffee (*F*[2, 1163.97] = 11.04, *p* < 0.001, ${\eta }_{p}^{2}=0.008$). Black coffee was liked best (*M* = 8.30, SD = 1.91), significantly preferred over the coffee consumed with 1–2 added extra ingredients (*M* = 8.09, SD = 2.01; *t*(2371.1) = 2.58, *p* = 0.027, *r* = 0.05), and especially preferred over coffee with more than 3 added ingredients (*M* = 7.74, SD = 2.24; *t*(715.5) = 4.59, *p* < 0.001, *r* = 0.17). Moreover, the coffee with 1–2 added ingredients was significantly preferred over the coffee with more than 3 added ingredients, with the latter having the lowest preference scores (*t*(665.4) = 2.99, *p* = 0.008, *r* = 0.12). Finally, the Number of coffees consumed per day significantly affected coffee preference (*F*[2, 348.41] = 24.22, *p* < 0.001, ${\eta }_{p}^{2}=0.015$). Participants who declared drinking 3–5 cups per day offered the highest liking scores (*M* = 8.44, SD = 1.90), reporting that they liked coffee significantly more than those consuming only 1–2 cups per day (*M* = 7.91, SD = 2.03; *t*(2313.4) = −6.91, *p* < 0.001, *r* = 0.14).

See Supplementary Information for further exploratory information-theoretic data analysis approaches (Table S2, Figs. S3, S4). Next, the complementary regression results for coffee Liking prediction are presented.

### Fractional response logit (FR) results to predict the overall coffee Liking score

**Fig. 4:**
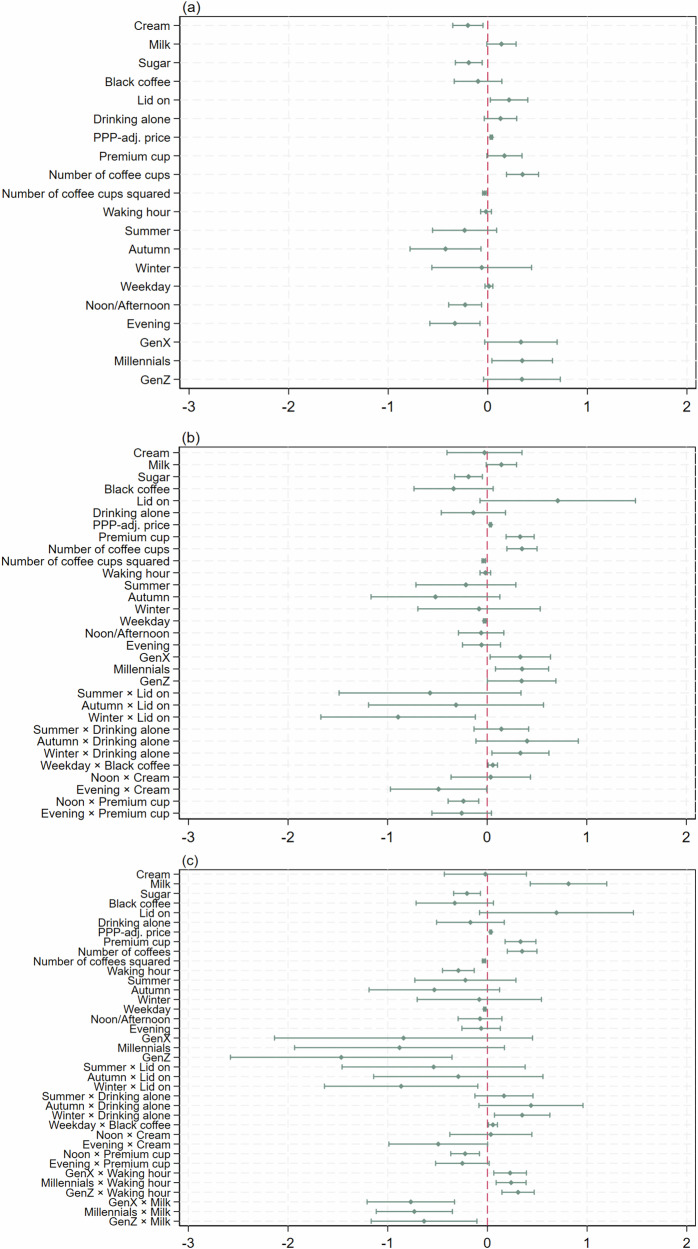
Predictors of coffee liking in the three fractional response (FR) regression, clustered by recruitment country. **a** Results from the Baseline model, **b** Results from the Temporal interaction model, and **c** Results from the Demographic interaction model. Diamonds represent estimated average beta coefficients, with errors bars denoting 95% CIs. Negative coefficients indicate coffee dislike. The red vertical line indicates statistical significance, i.e., any depicted error bar which crosses the red line is statistically non-significant. *N* = 2411.

FR coefficients and the average marginal effects (AMEs) for the three hierarchical fractional response models (i.e., the Baseline FR, the Temporal FR, and the Demographic FR) are presented in and Fig. 4 below, with supporting data in Tables S3, S4 in Supplementary Information.

The FR results indicate consistent patterns related to product-specific and habits in this dataset. Interestingly, sugar is associated with a statistically significant decrease (i.e., 3.1 percentage points) in predicted coffee Liking. To investigate potential reverse causality/selection concerns in the association between sugar and coffee liking, we apply Oster’s^22,23^ proportional-selection sensitivity analysis, which quantifies how strong unobserved selection would need to be to eliminate the association. We estimated two linear regressions: a model that focused on demographics, consumption context and the baseline full model with standard errors clustered by recruitment country. With ${R}_{\max }=\min \left(1,\,{1.3{xR}}_{{full}}^{2}\right)=0.094$, $\widetilde{\beta }{\rm{at}}\delta =1$ of −0.022, the association was highly robust to omitted-variable bias, with δ = 2.92 implying that unobserved selection would need to be nearly three times as strong as observed selection, so as to eliminate the estimated association. Higher PPP-adjusted prices are also related to higher reported liking, with each additional price unit associated with a 0.6 percentage point increase in liking scores. Habits-wise, each additional daily coffee consumed is associated with a 3.1 percentage points increase in coffee liking ratings. Note that the results suggest an optimal consumption level: Participants consuming just one cup of coffee per day report a relatively low liking score. As coffee consumption increases, so does satisfaction, peaking at five cups per day, that is, a 12.5% increase in Liking between one and five cups. However, beyond this optimal point, the coffee liking begins to drop, such that between five and ten cups, reported coffee liking decreases by 14.8%. These findings suggest that consumers who enjoy their coffee most drink around five cups/servings of coffee per day; see Fig. S5 in Supplementary Information.

Taking spring as the reference season, the Temporal FR results indicate that coffee liking significantly decreases by 6.4 percentage points in autumn. Moreover, while the use of a lid is associated with an overall increase in reported liking (+3.1 percentage points), this association is season-sensitive within this dataset. That is, relative to spring, the presence of a lid is associated with a significantly more pronounced seasonal decrease in liking scores, with 8.5, 7.9, and 10.4 percentage points decrease for each of the summer, autumn, and winter coffee liking assessments. Further, for participants drinking coffee in company, an additional statistically significant autumn penalty is observed, with reported coffee liking 8.6 percentage points lower than spring scores.

Further, compared to morning coffee consumption, coffee liking is 3.6 percentage points lower in noon/afternoon, and 4.6 percentage points lower in the evening. These results indicate that, in this sample, coffee liking scores were highest during the morning, with evening association varying significantly, depending on additives: While cream generally had a negative association, drinking coffee with cream in the evening was associated with a 13.2 percentage points decrease in predicted liking. This suggests a significantly lower liking for coffee with cream during late-day consumption in this sample.

The passage of the week is associated with a gradual erosion of enjoyment for those coffee drinkers who add ingredients to their coffee: Each additional day of the week is associated with a significant decline in coffee liking (i.e., −0.4 percentage points per day), representing a cumulative decrease of approximately 2.4 percentage points from the start to the end of the week. In contrast, the non-significant association between black coffee and weekday, suggests that additive-free coffee enjoyment appears to be less sensitive to temporal shifts.

Further, while consuming coffee from *premium* cups (i.e., ceramic and glass) is associated with an overall increase of 2.8 percentage points in coffee liking as compared to drinking coffee in regular cups (i.e., including plastic, paper, and metallic cups), Temporal FR results reveal that this advantage is time-sensitive: the drop from the morning high liking is steeper for the premium cup. Specifically, for participants using premium cups, liking significantly declines as the day progresses, with ratings 4.5 percentage points lower at noon, and 5.6 percentage points lower in the evening, as compared to the morning baseline. This diurnal decline is nevertheless absent for participants using regular cups for their coffee. This indicates that the positive association between premium receptacles and liking scores is a morning-specific phenomenon in our analysed sample.

Lastly, the Demographic FR results indicate that coffee is liked best by Gen X, Y, and Z, with coffee liking scores ranging from 9.0 to 9.8 percentage points higher, as compared to Baby Boomers. Further, the results indicate that the considered coffee additives are generation-sensitive. Specifically, Gen X, Millennials, and Gen Z participants report about 17% higher coffee Liking ratings as compared to Boomers when drinking their coffee *without* adding milk. However, this generational difference disappears when milk is added to the coffee. As such, in this sample, adding milk to the coffee is associated with statistically similar evaluations across the four generational cohorts, in contrast to the differences observed in non-milk-based coffee choices. Furthermore, waking hour associations were most pronounced among Boomers. With every additional hour added to a Boomer’s waking time, predicted coffee liking decreases by 5.7 percentage points. Millennials show a similar, though smaller, negative association (i.e., −0.9 percentage points), whereas Gen X and Gen Z coffee enjoyment scores remain statistically stable, irrespective of their waking hour.

### Zero-One Inflated Beta (ZOIB) results for predicting the perfect coffee Liking score

**Fig. 5:**
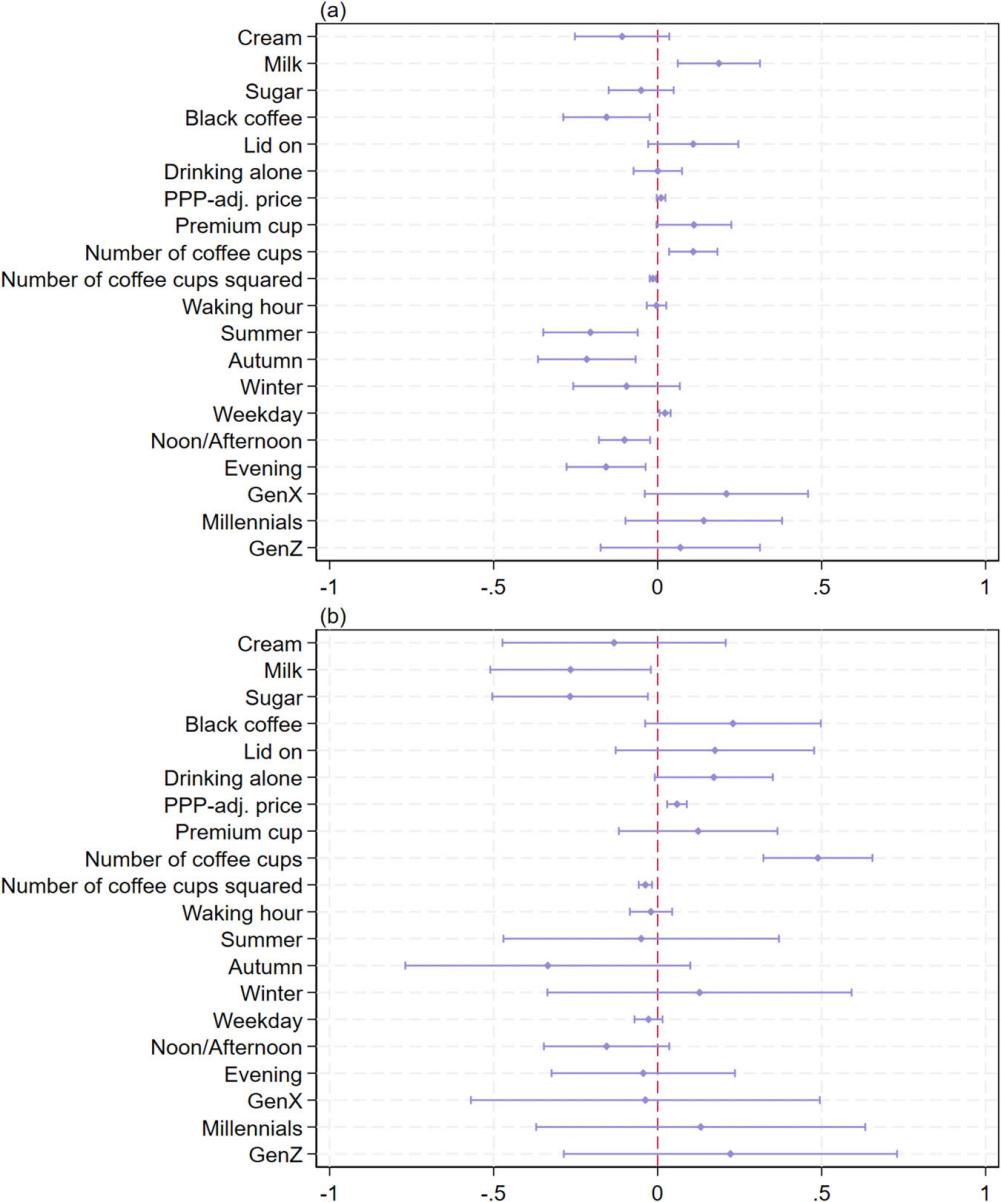
Zero-One Inflated Beta (ZOIB) results. **a** Factors influencing conditional mean coffee liking scores, i.e., 0 < rescaled coffee liking < 1, and **b** Factors influencing the probability of a perfect coffee liking score, i.e., rescaled coffee liking = 1. Diamonds represent estimated average beta coefficients, with errors bars denoting 95% CIs. Negative coefficients indicate coffee dislike. The red vertical line indicates statistical significance, i.e., any depicted error bar which crosses the red line is statistically non-significant. *N* = 2466.

Figure 5 presents the coefficients of the ZOIB model, with the supporting estimated marginal effects presented in Table S5 in Supplementary Information.

The results indicate that adding milk to coffee is associated with a significant increase in conditional mean coffee liking score (+3.9 percentage points), but nevertheless, a significant decrease in the probability of achieving a perfect coffee liking score (−5.6 percentage points). The number of cups of coffee consumed daily is associated with both model components, nevertheless this relationship is almost eight times greater for the probability of a perfect liking score (+6.2 percentage points), as compared to those reported for average liking ratings (+0.8 percentage points). That is, habitual drinkers identified in this dataset are significantly more likely to give a perfect coffee liking evaluation.

Black coffee, season (i.e., summer and autumn), and time of the day (i.e., noon, evening) are all associated with lower mean coffee liking scores, whereas the day of the week is associated with higher mean coffee liking scores. Furthermore, adding sugar is only associated with a significant decrease in the probability of a perfect score (−5.6 percentage points). Interestingly, price is not significantly related to mean liking levels in this dataset. Instead, price is only associated with the ceiling of expectations, with higher prices being associated with a 1.3 percentage point increase in the probability of a maximum liking score.

By comparing the results of the Baseline FR with those of the ZOIB, several relevant findings are evident: First, while a non-significant main effect for milk was found in the FR Baseline specification, the ZOIB results underline that adding milk to one’s coffee is in fact related to higher conditional mean coffee liking, as well as, importantly, to a significantly lower probability of giving a perfect coffee liking score. Second, while sugar was a consistent negative correlate in the FR model, the ZOIB once again underlines that adding sugar is especially associated with a reduced probability of giving a perfect liking score to one’s coffee. Third, the ZOIB model identifies a significant association between black coffee disliking and mean liking scores, a result that has not reach significance in the Baseline FR results. Fourth, the positive association for price and coffee liking found in the FR results appears to be related almost entirely to the probability of a maximum score in the ZOIB framework. Fifth, habitual coffee consumption was associated with coffee liking in both analysed models, but the ZOIB highlights that this relationship is much stronger for the maximum coffee liking ratings. Sixth, the penalties of time of the day and season found in the FR models appear to be related primarily to shifts in mean coffee liking within the ZOIB framework. Finally, the ZOIB analysis identified a weekday ascending trend for mean scores that was not observed in the FR results (see Table S5 in Supplementary Information).

### Robustness results in the geographical clustering by recruitment country

**Fig. 6:**
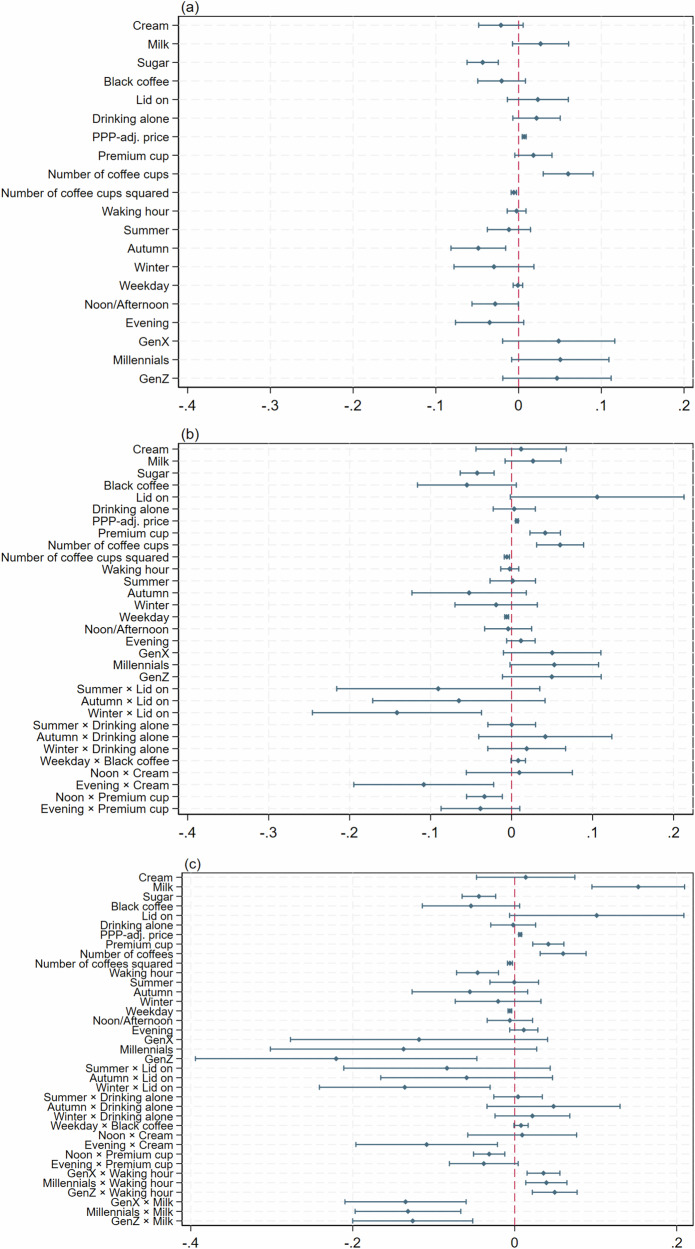
Predictors of coffee liking in the robustness multilevel linear mixed model (MLM). **a** Results from the Baseline model, **b** Results from the Temporal interaction model, and **c** Results from the Demographic interaction model. Diamonds represent estimated average beta coefficients, with errors bars denoting 95% CIs. Negative coefficients indicate coffee dislike. The red vertical line indicates statistical significance, i.e., any depicted error bar which crosses the red line is statistically non-significant. *N* = 2411.

See Fig. 6 for the multilevel linear mixed model (MLM) coefficients, with the supporting Tables S6, S7 in Supplementary Information, for the MLM results.

The MLM results identify a statistically significant, albeit small, country-level variance (${\sigma }_{u}^{2}=0.002$). This suggests that although participants’ initial ratings differed across the nine recruitment countries considered in the analysis, much of the variance (${\sigma }_{e}^{2}=0.044$) remained at the observation level within this dataset. As expected, switching from the FR model to a MLM specification resulted in an attenuation of most coefficient and marginal effects magnitudes.

Importantly, the MLM results highlight a shift of several attributes from consistent cross-national associations to context-specific associations. In this sense, even though the main effects for cream, lids, and evening were no longer independently significant in the MLM specification, their associations remained robust in specific contexts, see, e.g., the Cream × Evening and Season × Lid associations remained significant. Moreover, the only case in our sample where statistical significance was entirely lost within this random-intercept specification was the association for drinking alone (including its interaction with seasonality).

Nevertheless, the majority of predictors and interactions remained statistically significant. However, sugar, price and the interaction Season × Lid on registered more intense average marginal effects in the MLM as compared to the AMEs of FR model.

### Sensitivity analysis in support of the coffee Liking prediction

**Fig. 7:**
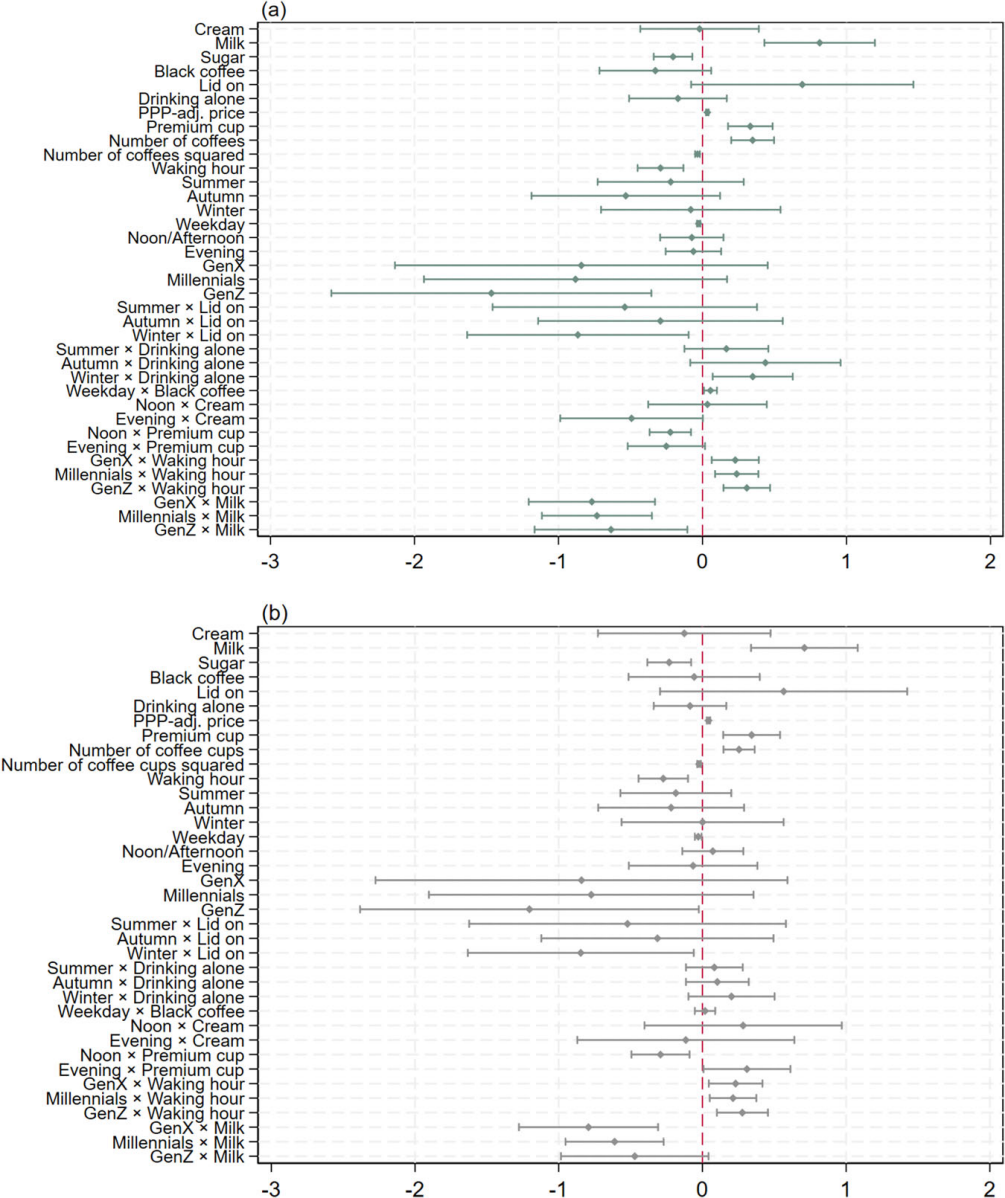
Predictors of coffee liking from the fractional regression (FR) model estimated in the sensitivity analysis. **a** Results on the full sample including Turkey, *N* = 2411, *AIC* = 2404.96; and **b** Results on the sample without the Turkey data, *N* = 1586, *AIC* = 1513.595. Diamonds represent estimated average beta coefficients, with errors bars denoting 95% CIs. Negative coefficients indicate coffee dislike. The red vertical line indicates statistical significance, i.e., any depicted error bar which crosses the red line is statistically non-significant.

To address the geographical imbalance of the sample, we re-estimated our FR model with clustered standard errors (see Fig. 7 below and the supporting Table S8 in Supplementary Information), after excluding the Turkish recruitment subsample (*N* = 871).

From the comparison of the two FR models, with/without the inclusion of Turkey data, it is interesting to note that several factors indicate higher average marginal effects (i.e., higher coffee liking) in the restricted sample. Specifically, the positive association between coffee liking and price, premium cup, and generation, was amplified. The negative association for sugar also became more pronounced and the main effect of waking hour reached statistical significance, suggesting that these factors of preference might be more intensely associated with coffee liking outside the Turkish contexts of consumption surveyed in this dataset. In contrast, the coefficients for habitual coffee consumption and season were attenuated in the model excluding the Turkish subsample. Similar reductions in the magnitude of AMEs were observed for interactions between Season × Lid on, Time of the day × Premium cup, Boomers × Waking hour, and Generation × Milk. Although these predictors remained relevant, the reduction in size could suggest that the strength of these specific associations in the full model was partially related to profiles characteristic of the Turkish data.

In conclusion, the sensitivity analysis confirms the stability of our analytical framework. Despite a 35% reduction in sample size undertaken for the sensitivity analysis, the majority of the primary predictors and interactions have maintained statistical significance. However, drinking coffee alone, the time of the day, and two interactions (Season × Drinking alone and Time × Cream) failed to reach significance in the smaller subset. It is nevertheless interesting to note that the noon/afternoon association and the Cream × Evening interaction remained significant within the MLM framework, confirming that these temporal effects are not artifacts of country-level clustering. The loss of statistical significance in the sensitivity analysis without Turkey data is rather very likely due to reduced statistical power, as opposed to a lack of generalisability. However, the drinking coffee alone main effect and its interaction with seasonality consistently lost statistical significance across both the multilevel specification and the model excluding the Turkish subsample, implying that these factors are more sensitive to country-level clustering and might be specific to cultural patterns within the Turkish data.

## Discussion

By taking a global citizen-science approach, coffee enjoyment was considered as a function of contextual, product, behavioural, and demographic factors. The study was conducted over a period of 11 months between April 2024 and February 2025, with 2987 coffee consumers voluntarily (i.e., without compensation) sharing their coffee experience in an ecologically-valid consumer setting, i.e., a naturalistic real-world setting, where one person would typically have a coffee, such as a coffee shop, one’s home, a park, or a student campus, to name a few. Importantly, while data collection took place in the Americas, Europe, Asia, and Australia, by collecting data in big metropolises (e.g., London, New York, Tokyo, Melbourne), our resulting dataset includes an international and truly diverse participant population. Our results highlight that coffee enjoyment peaks on spring mornings and coffee is liked best when consumed on Wednesdays. Season-wise, coffee ratings are lower in autumn compared to spring, and a similar penalty is observed for noon consumption relative to the morning consumption. Drinking coffee from premium cups (ceramic and/or glass) and higher prices are associated with higher coffee liking. Furthermore, drinking a coffee in the morning from a premium cup is significantly better liked than the same coffee type when consumed at noon. Further, the consumed coffee quantity shows diminishing returns, with the largest gains in liking identified for those drinking up to 5 cups per day, followed by a subsequent decline. The habit of waking-up later is linked to lower coffee liking and is concentrated among Boomers and Millennials. Drinking coffee with a lid on the cup during the colder seasons is associated with lower coffee liking, as does adding sugar to one’s coffee, and/or adding cream to coffee in the evening. Generational heterogeneity is evident in the evaluation of coffee with milk as a deciding factor: while Gen X, Millennials, and Gen Z participants reported significantly higher appreciation for milk-free coffee as compared to Boomers, this gap was attenuated when milk was added to the beverage. Collectively, the present findings sketch a robust profile for maximising coffee enjoyment, while highlighting substantial temporal and generational heterogeneity: moderate intake (4–5 cups/day) of higher-priced, unsweetened coffee, as well as, to ensure a maximum liking, keeping one’s coffee milk-free.

Intriguingly, across all statistical models, season emerges as closely linked to coffee enjoyment: That is, relative to spring, coffee liking declines in summer and even more in autumn. Autumn specifically lowers the probability of a perfect liking rating, with an evident shift from high to moderate coffee liking scores in the autumn. Seasonal shifts in liking may reflect changes in mood, individual preferences, sensory processing, or even sensory offerings. Although seasonal variations in food choice/flavour trends are well-documented^11,24,25^, evidence for seasonal changes in taste sensitivity remains limited. For example, only weak seasonal variation in the sensitivity to sweet and bitter have been documented^26^. In addition, shorter autumn daylight has been associated with “winter blues”, whereas spring may improve mood, a fact evident in coffee hedonic evaluations. Finally, caffeine’s effects may interact with these factors: At low doses, caffeine can improve hedonic tone, but at higher doses it increases tense arousal (i.e., anxiety, nervousness, jitteriness), potentially dampening the overall enjoyment^27^. Note though that the present results highlight that people’s consumption aligns with the specific recommended (healthy) amount of 3–5 cups/day^4,5^.

Robustness statistical checks preserve this pattern, with the mention that the summer liking penalty disappears, together with a slight attenuation in the autumn effect when the Turkish respondents are not included in the analysis. Overall, the seasonal impact is robust across tested model specifications and sample composition, yet heterogeneous by cultural context: Spring emerges as the most favourable season, whereas autumn is consistently associated with lower coffee liking, most notably among Turkish participants and across recruitment country clustering. Seasonal differences in people’s preferences have already been documented^11^.

In terms of timing, coffee is enjoyed most on Wednesdays, a result that hints that coffee may contribute to ease what Australians call a hump day – the hardest day of the week before getting closer to the week-end, or, similarly, as Germans put it—a ‘Bergfest’, a ‘party’ to celebrate the passage of the first half of the (working) week. In this context, it would be interesting to further explore whether Wednesday would also be a liking-day in other gastronomic settings—To the best of our knowledge specific weekday(s) haven’t been highlighted in relation to food liking, although research has opposed weekdays to weekends for daily energy intake (e.g., weekends/Saturday as most unhealthy diet^28^), or eating disorders^29^. Average coffee liking in this sample increases as the week progresses, aligning with the documented weekend effect in subjective evaluations: Research shows that people are likely to make more enthusiasm-based judgments when assessing life satisfaction on weekends, as opposed to weekdays^30^, and this affective shift specifically increases ratings for hedonic products^31^. Furthermore, the time of day when consumption occurs relates to coffee enjoyment, with a clear morning advantage that aligns with established consumption habits and physiological effects (see also ref. ^32^). Morning coffee is linked to greater alertness, concentration, and positive mood and well-being^33^. In addition, empirical evidence indicates that a morning-type rather than all-day coffee-drinking pattern is associated with lower risks of all-cause mortality relative to non-drinkers^6^. The results reported here also suggest that coffee liking declines at noon and in the afternoon. Even more, a lower likelihood of average coffee ratings at noon/in the afternoon and evening was demonstrated. Late-day coffee drinking can suppress melatonin and perturb the circadian rhythm of sympathetic activity which may, in turn, diminish hedonic appreciation^33^. Further, Kanjanakorn and Lee^34^ found that morning and afternoon coffee consumers exhibited high-energy positive affect as well as greatest affect changes, while the evening group consumers appeared the most content, peaceful, and rested, all positive low-energy feelings. By contrast, cultural background influences the coffee liking effect magnitude: Time of day effects attenuate when accounting for country clustering and Turkish participants are excluded, the evening association vanishing, results consistent with the evidence that coffee occasions and contexts vary with traditions and cultural norms^9^. As such, the social dimension of a coffee break as a ritual with a strong relational and communication value is highlighted, with this effect already particularly evident in established cultures, such as Italy^35–37^. Taken together, our results indicate that morning (particularly in spring) constitutes one of the most favourable contextual combinations for coffee enjoyment, whereas noon (particularly in autumn) constitutes one of the least favourable.

Cup lids appear to be consistently linked to lower coffee enjoyment across seasons even more, with the lid penalty found to be largest in winter. This result aligns with evidence that a substantial share of flavour perception (estimated at 75–95%) arises from olfactory receptors in the nose^38^. By restricting aroma release, lids attenuate experiencing the full taste of the most popular, or liked, of food/beverage aromas^39^. The results of this research extend prior work by demonstrating a lid-by-season interaction. We also find that premium cups (ceramic, glass) are positively associated to coffee liking relative to plastic, paper, or metal cups, thus confirming previous evidence^15,16^. Note that these effects are amplified when Turkish participants data are included in the analysis, suggesting that cultural context has an impact on the magnitude of the effect^40^. The premium cup advantage disappears when coffee is consumed at noon, as compared to mornings. The morning coffee ritual is well-documented in the literature^41^, and with the drinking receptacle as a necessary part of the multisensory coffee drinking experience^15^, at noon, the coffee consumption related to a snack or a break or an after-lunch experience often has more of a social dimension (i.e., creating a moment to exchange with other persons^42^).

These results further indicate that adding cream/creamer in the evening and/or adding sugar to one’s coffee is associated with a lower overall liking and the lower likelihood of a perfect coffee rating. This result seems counterintuitive, given the evidence that coffee-flavoured beverages with higher sugar and medium-high milk content tend to be most liked^43,44^. However, Cornelis and van Dam^45^ demonstrate that the psychostimulant effects of caffeine outweigh the bitterness of caffeine. Nevertheless, the added-sugar-less-liked-coffee effect found in our data may simply reflect the well-known preference for the sweet taste: People will add sugar to their coffee to reduce the *expected* coffee bitterness. Importantly, these effects are heterogeneous. Note though that our sample reflects the population of coffee drinkers, as data collection took place in coffee-drinking environments. Participants who gave less than perfect ratings display a stronger aversion to black coffee. Moreover, the addition of milk is associated with higher mean liking scores, while simultaneously reducing the probability of achieving maximum liking ratings. Milk fat may act as a protective layer against black coffee aversion, by altering bitterness through potential partitioning mechanisms^46^. Relatedly, the enjoyment of coffee with added ingredients declines progressively throughout the week, whereas black coffee preferences remain stable. Indeed, when people are cognitively depleted, such as might happen as the workweek progresses, enjoyment of complex-flavoured foods drops^47^. In addition, a generational difference is observed in the evaluation of coffee consumed without milk, with Gen X, Millennials, and Gen Z reporting significantly higher coffee appreciation as compared to Boomers. Nevertheless, the addition of milk evens out coffee preference across all generational cohorts. Interestingly, adding milk is associated with an increase in the average reported coffee liking, as well as with a lower likelihood of a perfect liking rating for the consumed coffee.

In accordance with the well-known price–quality signalling relationship, our findings highlight that higher price associates with greater coffee enjoyment^48^, particularly by increasing the likelihood of awarding perfect liking scores to the consumed coffee, at least when consumers know the price (e.g., blind tests do not necessarily favour expensive wines^49^). Whereas our data links coffee enjoyment with higher-priced (typically speciality) coffee, note that expensive wines also appear to be eliciting a negative-type of affect^50^, suggesting that the specific drink consumed determines (the direction of) the price-quality effect. Coffee enjoyment is also modulated by contextual factors. Consider, for example, the tempo of music playing in the background^51^ or even the coffee size (e.g., the Starbucks coffees have 3–4 times the size of a regular coffee consumed elsewhere). Neuroimaging evidence supports an expectation-based (marketing placebo) mechanism. For example, two samples of the same wine result in a greater experienced pleasantness for the higher priced one (see ref. ^49^, for a review). It has been argued that the decision-making and motivation brain centres play a pivotal role in such price biases, with the medial prefrontal cortex appearing to integrate price information into evaluative judgements, and the ventral striatum—central to reward and motivation—tracking the hedonic experience^52^. Taken together, a higher price acts as an indicator of quality that increases enjoyment on average, but how this indicator works differs depending on the outcome (overall liking ratings versus perfect liking ratings). Such findings are especially relevant in the context of current increase in coffee prices^53,54^ that may determine a further (and yet novel) shift in customers’ purchasing and enjoyment of the much-appreciated drink^55^.

Our finding that coffee liking is positively linked to the number of cups consumed (but, nevertheless, with diminishing returns beyond 5 cups) is in line with the established theory and evidence and official dietary guidelines. Such a result is an exemplification of the Economics law of diminishing marginal utility, which states that each additional unit of a good yields less added satisfaction. The first cup of coffee thus provides the largest enjoyment boost, with each subsequent cup adding less enjoyment. In fact, this pattern is consistent with the safety guidance: The European Food Safety Authority (EFSA) Panel on Dietetic Products, Nutrition and Allergies^56^ posited that habitual caffeine intake of up to 400 mg/day does not raise safety concerns, whereas higher intakes can provoke anxiety, restlessness, and sleep disruption, effects that could curb additional enjoyment from the consumption of extra coffee. Moreover, heavy coffee consumption was associated with a 4% reduced likelihood of sustained happiness over time, whereas moderate intake was associated with a slightly higher likelihood of sustained optimism^57^. Notably, the present study association between daily coffee consumption and coffee liking is nearly eight times stronger for perfect liking scores, as compared to less-than-perfect ratings attributed to momentary consumed coffee.

Finally, our analysis reveals a link between waking time and coffee enjoyment, with generation-specific patterns. In general, earlier rising is associated with higher coffee ratings. Among Baby Boomers, each additional hour of sleep corresponds to roughly a 6 percentage points decline in overall liking, whereas among Millennials, later wake-ups reduce the probability of assigning a perfect rating by about 1 percentage point. Note that the present results link the habitual pattern of waking up at a later time to an identified decline in coffee liking; the specific waking hour for the day when the sampled coffee was consumed was not recorded/analysed here. Nevertheless, these findings are consistent with evidence that the so-called *morningness* increases with age and is positively related to well-being^58^, suggesting that alignment between one’s circadian preference and coffee-drinking routine may facilitate more favourable hedonic evaluations.

Several potential limitations of the present study must be acknowledged. First, our ecologically-valid citizen-science approach is based on self-report, potentially susceptible to reporting biases. Second, the urban provenience largely predominant in our dataset may limit the generalisability of our results to other rural and/or less industrialised contexts. Third, even though observed patterns identify significant associations between product-specific, habits, demographic, and contextual factors and coffee liking within this sample and are strongly supported by theory, the cross-sectional nature of the data limits the possibility of inferring causality. Further, with acknowledgement of the important selection of factors involved in coffee liking in the present study, further specialised sensory characteristics of the consumed coffee itself (e.g., roast profile, aroma intensity, acidity, origin) were not experimentally controlled, and thus leave open questions about how specific intrinsic coffee attributes interact with diverse extrinsic contextual variables. Finally, participants’ psychological states, relevant/not for coffee consumption (e.g., mood, stress, fatigue) were not assessed in the study, and they may have impacted the overall coffee liking experience.

Taken together, the present study presents a comprehensive investigation of the diverse factors associated with the enjoyment of a cup of coffee, including consumer and coffee characteristics, typical habits in relation to the consumed coffee and the context in which coffee is consumed. Considering the large multi-country coffee consumer population that contributed to the present conclusions, the present work highlights a multi-dimensional expression of those specific factors that make us enjoy a cup of coffee, as these are collectively influenced by psychology, culture, and economic context, and by the coffee itself. Building on our results, future research needs to integrate longitudinal ecological momentary assessment personalised designs to capture fluctuations in coffee enjoyment across time and contexts. As a theoretical bridging between the economics of utility, crossmodal perception, and (chrono-)nutrition, the enjoyment of a cup of coffee could thus be further modelled as a function of the *dynamic* interaction between temporal (e.g., time of day, season, weekday), sensory (e.g., flavour, aroma, temperature), behavioural (consumption habits), and any further psychosocial determinants (e.g., traditions, norms), with consideration of any known and relevant cross-beverage consumption insights (e.g., tea, chocolate, wine).

## Methods

### Participants

An a priori power analysis based on a logistic regression approximation (OR = 1.5, α = 0.05, power = 0.95, baseline probability = 0.30, *π* = 0.80) indicated a minimum sample of *N* = 1807 (G*Power 3.1^59,60^). An additional *post hoc* power analysis based on logistic regression confirmed that our sample provides a power of 0.90 to detect the observed effects, exceeding the conventional threshold for adequate statistical power (0.80). A total of 3004 participants took part in this study voluntarily. The sample included 1212 male participants (mean age of 35 years, SD = 13 years, age range 14–83 years), 1770 female participants (*M* = 30 years, SD = 11, age range 12–78 years), and 22 participants who did not declare their gender (*M* = 31, SD = 12, age range 19–68 years). Gender does not moderate the association between coffee preferences and the examined predictors in this study. Data from several individuals were excluded during data pre-processing, such that the final dataset considered in the analysis comprised data from 2987 validated respondents. The dataset was refined by excluding those participants with non-numerical reported wake-up times, typically waking up outside the 4–11 a.m. range (*N* = 2). Further, taking into consideration participants’ reported price paid for their coffee, we excluded those participants having paid more than 30 international dollars (PPP-adjusted, *N* = 15); see Figure S1 in Supplementary Information for the price distribution highlighted outliers. The participants were recruited via flyers presented to the clients of various coffee shops (see Supplementary Information for a list of the coffee shops and all the nationalities reported in the study) as well as through notices placed across several university campuses, in the vicinity of coffee shops (convenience sampling; see Table 1 for responder characteristics). Participation was not limited to one single time/cup of coffee. However, we consider the likelihood of participants submitting multiple responses to our survey exceedingly low. Participants were not paid for their involvement. The study was approved by the Ethics Board of the Alexandru Ioan Cuza University of Iasi, Romania (no. 245/09.02.2024). This study conforms to the Declaration of Helsinki and to all subsequent amendments (Declaration of Helsinki, 1964, 2013).

### Apparatus and materials

The study was based on a 3-minute survey created in Google Forms, sent via various adverts and QR-codes, and subsequently completed by our participants on their personal mobile devices. The survey first introduced participants to the scope of the study and data handling. Participants were also informed that the survey questions should be answered while drinking their current coffee. Once they gave their informed consent, the participants were presented with three sets of questions: **(I)** the Demographics section, including questions on age, gender, location (reported as city, coffee place), nationality, and whether they were a coffee professional (e.g., barista, roaster)t; **(II)** the Current Coffee section, including questions on diverse extrinsic qualities regarding the current coffee (e.g., whether the coffee was consumed while sitting, standing, or while walking, alone or with at least one other person, or whether the coffee cup had a lid on), intrinsic qualities of the present coffee (e.g., whether the coffee was consumed black, decaffeinated, and/or with the addition of either sugar, milk, cream, ice, caramel, or cocoa), the coffee price (open answer), coffee Liking (visual analogue scale, VAS, from 1 not at all to 10 very much), time of day when the coffee was consumed (morning, noon/afternoon, or evening, after 6 p.m.), and the material from which the coffee cup was made (e.g., ceramic, glass, metal, paper, or plastic); **(III)** the Habits assessment section, including questions surveying responders’ typical waking time (e.g., with responses from 4 a.m. to 11 a.m.), the amount of hours slept each night (e.g., 4 in hourly increments to 12 h), together with several measured from 1 (*very rarely*) to 10 (*very often*) VAS questions individually-targeting specific coffee-related habits (e.g., drinking decaffeinated coffee, drinking coffee while lying in bed, by slurping it, on the go (standing), or sitting), responders’ usual choice from intrinsic coffee qualities (e.g., it was consumed black, decaffeinated, and/or with the addition of either sugar, milk, cream, ice, caramel, cocoa), the number of coffees consumed each day (e.g., from 1 to 10), and the coffee they enjoyed most (e.g., the first coffee of the day, the after-lunch coffee, the after-dinner coffee, or the coffee consumed during a break). A separate question interrogated respondents as to whether they considered themselves more of a ‘coffee’ or a ‘tea’ person. One final question presented participants with a choice of several coffee cups and asked them to choose the one they considered best for drinking coffee. See the English version of the questionnaire at the following link: https://forms.gle/ezB3FiMhHZUaUp3w9.

### Procedure

The survey was designed to assess several open questions with respect to demographics and habits around coffee consumption (see the Materials section). Google translate was used to translate the original English language questionnaire into several other languages (i.e., Italian, Japanese, Brazilian Portuguese, Romanian, Spanish, and Turkish). Several native speakers verified and adjusted the resulting translations for exact meaning and their similarity to the original text. A Google Form survey was created for each language of the questionnaire, assuring equivalence in all details of the survey. A visual designer created the visual images of the cups used in the last question of the questionnaire, as well as a flyer/poster advert, which was used for promotion in each of the used languages.

Several coffee shops were contacted and agreed to promote our study amongst their clients. A convenience sample of coffee venues in countries covering both the Northern and Southern hemisphere was selected, as well as both coffee producer and consumer countries. The study includes the data collected between April 2024 and February 2025. See Supplementary Information for a list of participating coffee shops.

### Data analysis

The collected dataset was split according to the experimental questions. For the purposes of the present study, the focus falls on participants’ current coffee appreciation. The considered dataset comprises 29 explanatory variables, including: **a)** the individual socio-demographic characteristics (e.g., age, generation, gender, nationality, country development, type of country—coffee producer/not, type of consumer—regular/professional coffee worker); **b)** reported characteristics of the coffee consumed while filling in the study questionnaire (intrinsic qualities [e.g., cocoa, caramel, cream, decaffeinated, ice, milk, sugar, black coffee]; total intrinsic qualities [i.e., total added ingredients], Purchasing Power Parity (PPP)-adjusted price, type of cup), **c)** several general coffee consumption habits (extrinsic qualities [e.g., lid on/not, consumed alone, consumed with company, while sitting, while standing, while walking]), and **d)** several general consumer and participants’ sleep-related variables (e.g., season, time of day, weekday, wake-up hour, hours of sleep, reported number of coffees consumed per day).

### Information-theoretic data exploration

One of the first steps taken during data exploration was to visualise data and to evaluate the importance of each variable in the proposed dataset for the prediction of coffee Liking. As such, an information-theoretic approach was used, based on entropy and measures of information gain^61^.

To compute the entropy, the variable Liking was discretised into six categories: *low liking* (1–4)—6.41% of responses, *medium liking* (5-6)—9.74% of responses, *medium-to-high liking* (7)—12.96% responses, *high liking* (8) —21.99% responses, *very high liking* (9)—16.12% responses, and *perfect liking* (10)—32.77% responses.

Specifically, to compute entropy, for ${{Liking}}_{i}$ with the identified 6 possible states, each having the corresponding probability of $p({{Liking}}_{i})$, the average amount of information gained from the component measurement (${{Liking}}_{i}={{Liking}}_{1},\ldots ,{{Liking}}_{6})$, entropy ${H}_{S}\left({Liking}\right)$ was calculated considering Shannon’s formula^62^ as in Eq. 1:

1 $${H}_{S}\left({Liking}\right)=-\mathop{\sum }\limits_{i=1}^{6}p({{Liking}}_{i}){\log }_{2}[p({{Liking}}_{i})]$$

Additionally, for each $X$ predictor variable from the dataset, we calculated the corresponding conditional entropy in Eq. 2:

2 $$H({Liking}|X)=\mathop{\sum }\limits_{j=1}^{m}p({x}_{j})H({Liking}|X={x}_{j})$$

Where *m* is the number of distinct values of predictor X, $p({x}_{j})$ is the probability of $x$ taking value ${x}_{j}$, $H({Liking}|X={x}_{j})$ is the entropy of Liking given $X={x}_{j}$.

Further, information gain (IG) was computed to identify those variables from the dataset that provided the most meaningful reduction in uncertainty when predicting individual coffee liking scores, with the formula in Eq. 3:

3 $${IG}\left({Liking},X\right)=H\left({Liking}\right)-\,H({Liking}|X)$$

Note that while IG provides a useful measure to assess a variable’s importance, it tends to favour those variables discretised in many values/categories. With this consideration in mind, we further derived, for each variable, the gain ratio, by normalising the IG by the entropy of the predictor variable itself. Specifically, the gain ratio is defined as in Eq. 4:

4 $${GainRatio}({Liking},X)=\frac{{IG}\left({Liking},X\right)}{H\left(X\right)}$$

Where $H\left(X\right)$ is the entropy of a given predictor variable $X$, also known as split information.

### Group-based data exploration

In a second pre-processing step, for each of the considered variables (i.e., generation, season, weekday, time of day, type of coffee cup, added ingredients, number of coffees consumed), one-way Welch’s analyses of variance (ANOVAs) were conducted to identify any differences in coffee preferences across groups (alpha level of 0.05 was selected). Partial *η*^2^ was used as effect size for the one-way ANOVAs. Levene’s test of equality of error variances was used to assess homogeneity of variances and any identified significant main effects were followed-up with Games-Howell post-hoc tests.

### Complementary regression approaches to predict coffee Liking

In the next data analysis steps, the modelling strategy used a sequence of complementary regression frameworks, including: ***(1)*** a series of fractional response (FR) models for prediction in a hierarchical framework, with a logit link for the conditional mean for coffee Liking, and ***(2)*** a Zero-One Inflated Beta (ZOIB) model, to determine if the drivers of coffee Liking are uniform across the used scale. These models were followed by two control analyses, with ***(3)*** a robustness analysis using linear mixed-effects model with random intercepts for recruitment countries, to account for any spatial dependencies and structural recruitment imbalances, and ***(4)*** a sensitivity analysis where the primary FR models were re-estimated with the exclusion of the dominant geographical subsample (Turkey), to verify the stability of our findings. Specifications for each of the performed models are further detailed below:

1. To determine the factors that influence the overall level of coffee preference, the data analysis started by estimating a FR model. The dependent variable, coffee Liking, is measured on a bounded scale from 1 to 10 and has a high concentration of observations at the upper extreme: Specifically, coffee Liking is significantly left-skewed, with 70.88% of values at 8 or higher, and 32.77% at the maximum value of 10; see Fig. 1. This observed distributional pattern classifies it as a corner solution response, where values are non-negative, follow a roughly continuous distribution over the positive range, yet are strongly clustered at the upper boundary^63^. Skewness gives high-leverage observations and increases the likelihood of nonlinear relationships between explanatory variables and coffee Liking^64,65^. Not considering the continuous, yet bounded, highly skewed and non-linearity specific of the data may lead to biased parameters and sensitivity to distributional misspecification^66,67^. Then, to achieve robustness against potential distributional errors, a quasi-likelihood estimation approach warrants the consistency of coefficient estimates, irrespective of the true underlying conditional mean distribution^68^.
  As such, the coffee Liking scores were re-scaled to fit within the required 0-1 range with the transformation Liking = (Liking − 1)/9, while preserving the dependent variable relative distribution and allowing it to be modelled as a bounded outcome. By consequence, the 1–10 Liking scale is considered in the analysis as a proportion of the maximum possible score or preference intensity. In this context, the FR logit model for coffee Liking across full preference spectrum can thus be formally expressed as in Eq. 5:
  5 $${\rm{E}}({\rm{y}}|{\bf{x}})={\rm{G}}\left({{\rm{x}}}_{{\rm{i}}}{\boldsymbol{\beta }}\right)\,\forall {\rm{I}}$$
  With the response variable, the transformed Liking score (scaled and considered in the [0,1] range), $G\left(.\right)$ is the logistic cumulative distribution function with $0 < G\left(z\right) < 1,\,\forall z{\mathbb{\in }}{\mathbb{R}}$, $G\left(x\right)\equiv \Lambda (z)\equiv \frac{\exp (z)}{1+\exp (z)}$ and $G\left(x\right)\equiv \Phi (z)$, where $\Phi (.)$ is the standard normal cdf, ${x}_{i}=({x}_{i1},\ldots .{x}_{{ip}})$ is a $p\times 1$ vector that contains the values of the covariates, $\beta ={({\beta }_{1},\ldots ,\,{\beta }_{p})}^{{\prime} }$ is a $p\times 1$ vector of parameters to be estimated.
  To ensure the inclusion of all participants, robust standard errors are reported. Further, to account for the nested structure of the data, robust standard errors are used at the cluster level, by taking the country of recruitment as the clustering variable. This approach relaxes the assumption of independence between observations, recognising that within the same national market, consumers may share unobserved cultural, sensory, and/or environmental traits, that influence coffee preference. For the purpose of variance estimation, clustering was thus limited to the main nine recruitment countries where *N* > 30. This threshold was set to ensure sufficient variation within one considered cluster for the estimation of the variance-covariance matrix. As such, the FR logit models with both robust standard errors (*N* = 2466) and robust standard errors clustered by recruitment country (*N* = 2411) were included in the analysis of product-specific, habits, demographic, and contextual factors related to the reported coffee Liking. This analysis follows a hierarchical specification strategy that is used in all the reporting of the results, including: a *Baseline* specification, where we analyse the participant behaviour to answer our experimental question, i.e., *What makes us enjoy a cup of coffee?*, a *Temporal* specification, including interactions that explore how temporal factors are related to coffee liking, and a *Demographic* specification that addresses generation heterogeneity to investigate whether generational cohorts change the patterns.
2. To account for the clustering of observations at the highest score (Liking = 10), a Zero-One Inflated Beta (ZOIB) model^69^ was performed. The ZOIB regression accounts for the presence of mass points at 0 and 1 by allowing these extreme proportions to arise from processes distinct from those generating intermediate values^70,71^. The model consists of two logistic regressions to predict whether the proportion equals 0, *zero-inflate*, or 1, *one-inflate*, and one beta regression model to capture outcomes within the open interval (0, 1). This framework yields consistent and unbiased parameter estimates^72^. Given the significant ceiling effect, as compared to the relatively few floor responses found in the present dataset (i.e., 32.77% at Liking =10 vs. 1.43% at Liking=1), a full set of main predictors for the one-inflation component and the continuous beta distribution is specified. Further, to ensure model convergence and prevent overfitting of the rather rare zero-valued observations, the zero-inflation component is modelled only with the constant.
3. As a robustness check, a multilevel linear mixed (MLM) regression was estimated to further address potential convenience-based recruitment dependencies. For this, random intercepts at the country level were used, to account for unobserved heterogeneity across recruitment clusters. By applying the MLM to our hierarchical framework, the analysis assesses whether fine-grained contextual associations, such as time of day, weekday, or seasonal patterns remain robust when accounting for recruitment country clustering. The model specification is presented in Eq. 6:
  6 $${\bf{y}}={\bf{X}}{\boldsymbol{\beta }}+{\bf{Zu}}+{\boldsymbol{\epsilon }}$$
  Where ${\bf{y}}$ is the *n* × 1 vector of responses, ${\bf{X}}$ is an *n* × *p* design/covariate matrix for the fixed effects ${\boldsymbol{\beta }}$, and ${\bf{Z}}$ is the *n* × *q* design/covariate matrix for the random effects ${\bf{u}}$. The *n* × 1 vector of errors ${\boldsymbol{\epsilon }}$ is assumed to be multivariate normal with mean 0 and variance matrix ${{\rm{\sigma }}}_{{\rm{\epsilon }}}^{2}{\bf{R}}$.
4. Lastly, an additional sensitivity analysis was conducted to verify the robustness of our findings. Given the substantial representation of Turkish participants in the sample (*N* = 1058 in the full dataset), the main FR interaction model was re-estimated after excluding all data collected in Turkey (*N* = 871 observations with non-missing price), to assess the generalizability of the results.

Akaike Information Criterion (*AIC*) was used as a metric of model fit, with pseudo-R² reported for descriptive purposes. For each FR model, we report joint Wald tests for the factors, with Holm–Bonferroni corrections applied to main effects, and the Benjamini–Hochberg false discovery rate (FDR) reported for interaction blocks. Analyses were conducted using Stata/BE version 19.0 (STATA Corp., College Station, TX, US) and JASP (version 0.19.3^73^).

## Supplementary information

Supplementary information

## Data Availability

The dataset analysed in the current study is not currently publicly available. The present dataset is part of a larger dataset, parts of which are currently being prepared for publication in three other manuscripts, including one specific dataset manuscript focusing on the publishing of the data collected in the entire dataset. With the publication of the general coffee dataset, the dataset analysed in the present manuscript will be public and available from the corresponding author on reasonable request.

## Code availability

The underlying code for this study is not publicly available but may be made available to qualified researchers on reasonable request from the corresponding author.

## References

[1] Sánchez Guerrero, J. A. Top 10 coffee-producing countries in 2024. *Coolx*. https://coolx.earth/top-10-coffee-producing-countries-in-2024/ (2024).

[2] Triolo, F. A., Figueiredo, B., Martin, D. M. & Farrelly, F. Coffee: a global marketplace icon. *Consum. Mark. Cult.* **26**, 311–320 (2023).

[3] International Coffee Organization. *Coffee Market Report: Beyond Coffee – Towards a Circular Coffee Economy*. https://www.icocoffee.org/documents/cy2024-25/coffee-development-report-2022-23.pdf (2024).

[4] Barrea, L. et al. Coffee consumption, health benefits and side effects: a narrative review and update for dietitians and nutritionists. *Crit. Rev. Food Sci. Nutr.* **63**, 1238–1261 (2021). 34455881 10.1080/10408398.2021.1963207

[5] Di Maso, M. et al. Caffeinated coffee consumption and health outcomes in the US population. *A dose–response meta-analysis. Adv. Nutr.* **12**, 1160–1176 (2021). 33570108 10.1093/advances/nmaa177 PMC8321867

[6] Wang, X. et al. Coffee drinking timing and mortality in US adults. *Eur. Heart J.* **46**, 749–759 (2025). 39776171 10.1093/eurheartj/ehae871 PMC11843000

[7] Loftfield, E. et al. Coffee drinking is widespread in the United States, but intake varies by demographics. *J. Nutr.* **146**, 1762–1768 (2016). 27489008 10.3945/jn.116.233940 PMC4997286

[8] Khan, W., Sainger, G., Siddiqui, M. S., Mateen, B. Percolating insights: a study on coffee purchase behaviour in an emerging economy. *Br. Food J*. 10.1108/BFJ-01-2025-0039 (2025).

[9] Samoggia, A. & Riedel, B. Coffee consumption and purchasing behaviour review. *Appetite* **129**, 70–81 (2018). 29991442 10.1016/j.appet.2018.07.002

[10] Spence, C. Explaining diurnal patterns of food consumption. *Food Qual. Prefer.* **91**, 104198 (2021).

[11] Spence, C. Explaining seasonal patterns of food consumption. *Int. J. Gastron. Food Sci.* **24**, 100332 (2021).

[12] Deloitte Switzerland. *Coffee Report*. https://www.deloitte.com/ch/en/Industries/consumer/perspectives/coffee-study.html (2024).

[13] Wang, M. J., Opoku, E. K. & Tham, A. Exploring Gen-Z consumers’ preference for specialty coffee in Taiwan. *Young Consum.* **25**, 368–382 (2024).

[14] Kent R. Digital food tracking. In *Research Methods in Digital Food Studies* 143–163 (Routledge, 2021).

[15] Carvalho, F. M. & Spence, C. Cup colour influences consumers’ expectations and experience on tasting specialty coffee. *Food Qual. Prefer.* **75**, 157–169 (2019).

[16] Carvalho, F. M. & Spence, C. Do metallic-coated cups affect the perception of specialty coffees?. *Int. J. Gastron. Food Sci.* **23**, 100285 (2021).

[17] Labbe, D., Rytz, A., Strube, A. & Leloup, V. Impact of mug shape and beverage volume on instant coffee perception. *Food Qual. Prefer.* **89**, 104150 (2021).

[18] Vegro C. L. R., de Almeida L. F. Global coffee market: socio-economic and cultural dynamics. In *Coffee Consumption and Industry Strategies in Brazil* 3–19. 10.1016/B978-0-12-814721-4.00001-9. (Woodhead, 2020).

[19] Kurz, J., Efendić, E. & Goukens, C. Pricey therefore good?. *Psychol. Mark.* **40**, 1115–1129 (2023).

[20] Motoki, K., Takahashi, A. & Spence, C. Tasting atmospherics: Taste associations with coffee shop interiors. *Food Qual. Prefer.* **94**, 104315 (2021).

[21] Okop, K. J. et al. Multi-country citizen-science projects to co-design cardiovascular disease prevention strategies. *BMC Public Health* **23**, 2484 (2023). 38087240 10.1186/s12889-023-17393-x PMC10714547

[22] Oster, E. Unobservable selection and coefficient stability. *J. Bus. Econ. Stat.* **37**, 187–204 (2017).

[23] Spence C. *Gastrophysics: The New Science of Eating* (Viking Penguin, 2017).

[24] Spence, C. Ginger: The pungent spice. *Int. J. Gastron. Food Sci.* **33**, 100793 (2023).

[25] Spence, C. Nutmeg and mace: The sweet and savoury spices. *Int. J. Gastron. Food Sci.* **36**, 100936 (2024).

[26] Costanzo, A. Temporal patterns in taste sensitivity. *Nutr. Rev.* **82**, 831–847 (2024). 37558243 10.1093/nutrit/nuad097 PMC11082591

[27] Nehlig, A. Is caffeine a cognitive enhancer?. *J. Alzheimers Dis.* **20**, 85–94 (2010). 10.3233/JAD-2010-091315 20182035

[28] An, R. Weekend-weekday differences in diet among US adults. *Ann. Epidemiol.* **26**, 57–65 (2016). 26559331 10.1016/j.annepidem.2015.10.010

[29] Forester, G. et al. Time-of-day and day-of-week patterns of binge eating. *Int. J. Eat. Disord.* **56**, 1694–1702 (2023). 37212510 10.1002/eat.23995 PMC10600945

[30] Diener, E., Inglehart, R. & Tay, L. Theory and validity of life satisfaction scales. *Soc. Indic. Res.* **112**, 497–527 (2013).

[31] Du, J., Zhu, L., Ma, Y. & Zhang, Y. Beyond weekdays: The impact of the weekend effect on eWOM of hedonic product. *J. Retail. Consum. Serv.* **77**, 103624 (2024).

[32] Martyn, D., Lau, A., Richardson, P. & Roberts, A. Temporal patterns of caffeine intake in the US. *Food Chem. Toxicol.* **111**, 71–83 (2018). 29109041 10.1016/j.fct.2017.10.059

[33] Lüscher, T. F. Start your day with a morning coffee!. *Eur. Heart J.* **46**, 760–762 (2025). 39776142 10.1093/eurheartj/ehae823

[34] Kanjanakorn, A. & Lee, J. Examining emotions in coffee drinkers in natural environment. *s. Food Qual. Prefer.* **56**, 69–79 (2017).

[35] Olivetta, E. Starbucks from USA to Italy: Not only coffee. *Micro & Macro Mark* **3**, 489–508 (2017).

[36] Barmeyer, C., Mayrhofer, U. & Würfl, K. Informal information flows in organizations: The Italian coffee break. *Int. Bus. Rev.* **28**, 796–801 (2019).

[37] Bertolini, S. & Tosi, S. Distanziamenti e capitale sociale in smart working. *Meridiana* **104**, 101–124 (2022).

[38] Spence, C. Just how much of what we taste derives from smell?. *Flavour* **4**, 30 (2015).

[39] Spence, C. Leading the consumer by the nose: On olfactory design. *Flavour* **4**, 31 (2015).

[40] Jeong, S. & Lee, J. Effects of cultural background on consumer perception and acceptability of foods and drinks: a review of latest cross-cultural studies. *Curr. Opin. Food Sci.* **42**, 248–256 (2021).

[41] Ratcliffe, E., Baxter, W. L. & Martin, N. Consumption rituals relating to food and drink: a review and research agenda. *Appetite* **134**, 86–93 (2019). 30572007 10.1016/j.appet.2018.12.021

[42] Spinelli, S. et al. Investigating preferred coffee consumption contexts using open-ended questions. *Food Qual. Prefer.* **61**, 63–73 (2017).

[43] Li, B., Hayes, J. E. & Ziegler, G. R. Maximizing overall liking: a coffee-flavoured milk case study. *Food Qual. Prefer.* **42**, 27–36 (2015). 26005291 10.1016/j.foodqual.2015.01.011 PMC4438862

[44] Varela, P., Beltrán, J. & Fiszman, S. Uncovering drivers of coffee liking. *Food Qual. Prefer.* **32**, 152–159 (2014).

[45] Cornelis, M. C. & van Dam, R. M. Genetic determinants of liking and intake of bitter foods. *Sci. Rep.* **11**, 23845 (2021). 34903748 10.1038/s41598-021-03153-7 PMC8669025

[46] Li, B., Hayes, J. E. & Ziegler, G. R. Interpreting consumer preferences: physicohedonic and psychohedonic models yield different information in a coffee-flavored dairy beverage. *Food Qual. Prefer.* **36**, 27–32 (2014). 25024507 10.1016/j.foodqual.2014.03.001 PMC4094130

[47] Hadi, R., Rubin, D., Hildebrand, D. & Kramer, T. Flavor fatigue: How cognitive depletion influences consumer enjoyment of complex flavors. *J. Consum. Psychol* **31**, 103–111 (2021).

[48] Li, X. E., Jervis, S. M. & Drake, M. A. Extrinsic factors influencing product acceptance: a review. *J. Food Sci.* **80**, R901–R909 (2015). 25959688 10.1111/1750-3841.12852

[49] Spence, C. Cognitive influence on wine evaluation. *Food Res. Int.* **187**, 114411 (2024). 38763664 10.1016/j.foodres.2024.114411

[50] Souza-Coutinho, M. et al. Consumers associate high-quality wines with complexity and persistence. *Foods* **9**, 452 (2020). 32276305 10.3390/foods9040452 PMC7230440

[51] Yeoh, J. P. S. & Spence, C. Music to my lips: effects of musical tempo on coffee drinking. *J. Sens. Stud.* **40**, e70040 (2025).

[52] Schmidt, L. et al. How context alters value: neural links between price cues and taste. *Sci. Rep.* **7**, 8098 (2017). 28808246 10.1038/s41598-017-08080-0 PMC5556089

[53] Convery S. Why are coffee prices going up? *The Guardian* (7 Jan 2025). https://www.theguardian.com/australia-news/2025/jan/07/coffee-prices-australia-going-up-cafe-flat-white-cost.

[54] Wood Z. Coffee drinkers face price rises as costs hit record high. *The Guardian* (11 Dec 2024). https://www.theguardian.com/food/2024/dec/10/coffee-drinkers-face-price-rises-as-costs-on-global-markets-hit-record-high.

[55] Bryant M. ‘Fika has become more expensive’: Rising coffee prices affect a Swedish tradition. *The Guardian* (12 Apr 2025). https://www.theguardian.com/world/2025/apr/12/fika-sweden-coffee-prices.

[56] EFSA Panel on Dietetic Products, Nutrition and Allergies Scientific opinion on the safety of caffeine. *EFSA J.* **13**, 4102 (2015). 10.2903/j.efsa.2015.4148 PMC13151561 42109818

[57] Qureshi, F., Stampfer, M., Kubzansky, L. D. & Trudel-Fitzgerald, C. Coffee consumption and psychological well-being. *PLoS ONE* **17**, e0267500 (2022). 35679227 10.1371/journal.pone.0267500 PMC9182697

[58] Ágoston, C. et al. Morningness–eveningness and caffeine consumption: A path analysis. *Chronobiol. Int.* **36**, 1301–1309 (2019). 31216901 10.1080/07420528.2019.1624372

[59] Erdfelder, E., Faul, F., Buchner, A. & Lang, A.-G. Statistical power analyses using G*Power 3.1: tests for correlation and regression analyses. *Behav. Res. Methods* **41**, 1149–1160 (2009). 19897823 10.3758/BRM.41.4.1149

[60] Faul, F., Erdfelder, E., Lang, A.-G. & Buchner, A. G. Power 3: a flexible statistical power analysis program for the social, behavioral, and biomedical sciences. *Behav. Res. Methods* **39**, 175–191 (2007). 17695343 10.3758/bf03193146

[61] Shannon, C. E. A mathematical theory of communication. *Bell Syst. Tech. J.* **27**, 379–423 (1948).

[62] Shannon, C. E. Prediction and entropy of printed English. *Bell Syst. Tech. J.* **30**, 50–64 (1951).

[63] Wooldridge J. M. *Econometric Analysis of Cross Section and Panel Data* (MIT Press, 2010).

[64] Lee, C. S. & Conway, C. The role of generalized linear models in handling cost data. *Eur. J. Cardiovasc. Nurs.* **21**, 392–398 (2022). 35076072 10.1093/eurjcn/zvac002

[65] Manning W. Dealing with skewed data on costs and expenditures. In Jones A. M. (Ed.) *The Elgar Companion to Health Economics* 473–480 (Edward Elgar, 2012).

[66] Gallani S., Krishnan R. Applying the fractional response model to survey research. *Harvard Bus. Sch. Acct. Mgmt. Unit Working Paper* 16-016 10.2139/ssrn.2642854 (2017).

[67] Molenaar, D., Cúri, M. & Bazán, J. L. Zero and one-inflated IRT models for bounded continuous data. *J. Educ. Behav. Stat.* **47**, 693–735 (2022).

[68] Papke, L. E. & Wooldridge, J. M. Econometric methods for fractional response variables. *J. Appl. Econ.* **11**, 619–632 (1996).

[69] Ospina, R. & Ferrari, S. L. P. Inflated beta distributions. *Stat Papers* **51**, 111–126 (2010).

[70] Nappo, N., Fiorillo, D. & Lubrano Lavadera, G. Subjective job insecurity during the COVID-19 pandemic in Italy. *Ital Econ. J.* **9**, 1153–1179 (2023).

[71] Buis M. L. *Analyzing proportions*. Eighth German Stata Users Group meeting (Institut für Soziologie Eberhard Karls Universität Tübingen, 2010).

[72] Dangerfield, F. et al. Urban-regional patterns of food purchasing behaviour: a cross-sectional analysis of the 2015–2016 Australian Household Expenditure Survey. *Eur. J. Clin. Nutr.* **75**, 697–707 (2021). 32920603 10.1038/s41430-020-00746-9

[73] JASP Team. (2022). *JASP (Version 0.16.1) [Computer software]*.

[74] Watson, N. F. et al. Recommended amount of sleep for a healthy adult. *J. Clin. Sleep Med.* **11**, 591–592 (2015). 25979105 10.5664/jcsm.4758 PMC4442216

[75] Walch, O. J., Cochran, A. & Forger, D. B. A global quantification of normal sleep schedules using smartphone data. *Sci. Adv.* **2**, e1501705 (2016). 27386531 10.1126/sciadv.1501705 PMC4928979

[76] World Bank. *World Development Indicators* (accessed 2025).

