# Supplementary material for: A multi-country citizen-science study on what makes us enjoy a cup of coffee
Source: NPJ Sci Food. 2026 Apr 22;10:208. doi: 10.1038/s41538-026-00832-5 (PMC13323992; doi:10.1038/s41538-026-00832-5)
Supplement: Supplementary file 1 — Supplementary information [file 41538_2026_832_MOESM1_ESM.docx]

**SUPPLEMENTARY INFORMATION**


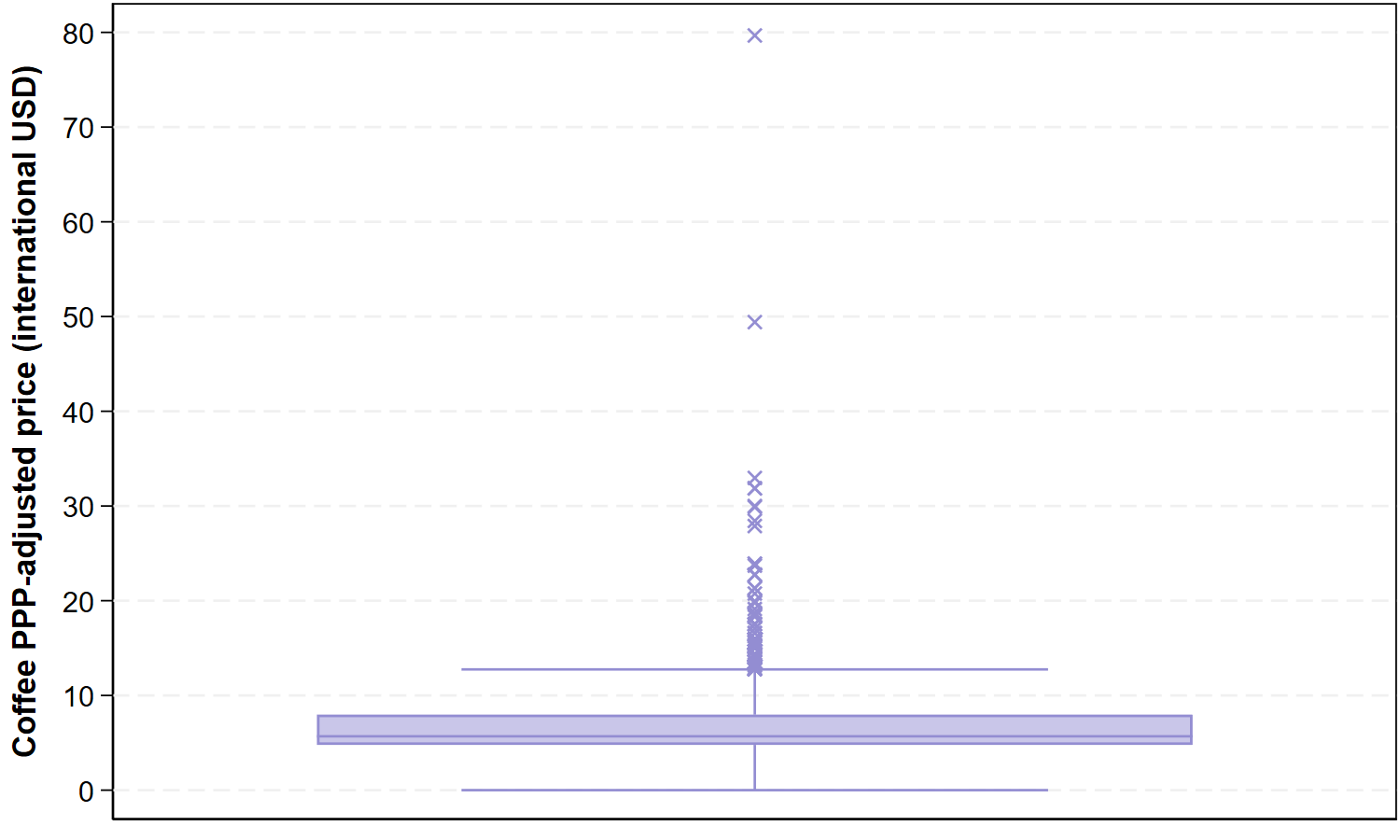


**Figure S1.** Boxplot of the price of a cup of coffee, *N* = 3,002. The box includes the middle 50% of the price data, the whiskers extend to the lower (bottom 25%) and higher quartile (top 25%) of data; the thick horizontal line inside the box represents the median. Identified individual data with values higher than 1.5 times the interquartile range are denoted with a x sign, as they may be considered potential outliers in the price distribution. Note the compressed y-axis; the 60–130 USD distance not shown to scale. *PPP* – Purchasing Power Parity.

**Table S1.** Distribution summary of recruitment country by month.

| Country | Apr24 | May24 | Jun 24 | Jul 24 | Aug24 | Sep 24 | Oct 24 | Nov24 | Dec24 | Jan 25 | Feb 25 | **Total** |
| --- | --- | --- | --- | --- | --- | --- | --- | --- | --- | --- | --- | --- |
| Argentina | 0 | 0 | 0 | 0 | 0 | 0 | 0 | 1 | 0 | 0 | 0 | **1** |
| Australia | 0 | 0 | 7 | 40 | 0 | 0 | 0 | 9 | 9 | 5 | 1 | **71** |
| Brazil | 0 | 67 | 5 | 3 | 2 | 0 | 0 | 0 | 0 | 0 | 0 | **77** |
| Canada | 0 | 2 | 0 | 1 | 1 | 0 | 1 | 0 | 1 | 0 | 0 | **6** |
| Colombia | 0 | 20 | 3 | 0 | 82 | 157 | 0 | 0 | 0 | 0 | 0 | **262** |
| Cyprus | 0 | 0 | 0 | 0 | 0 | 0 | 0 | 0 | 0 | 1 | 0 | **1** |
| Czech Rep. | 0 | 0 | 1 | 0 | 0 | 0 | 0 | 1 | 0 | 0 | 0 | **2** |
| Ethiopia | 0 | 0 | 0 | 0 | 1 | 0 | 0 | 0 | 0 | 0 | 0 | **1** |
| France | 0 | 0 | 0 | 1 | 1 | 0 | 0 | 0 | 0 | 0 | 0 | **2** |
| Germany | 0 | 0 | 0 | 0 | 2 | 0 | 1 | 0 | 2 | 1 | 0 | **6** |
| Hong Kong | 0 | 0 | 0 | 1 | 0 | 0 | 0 | 0 | 0 | 0 | 0 | **1** |
| India | 0 | 0 | 1 | 0 | 0 | 0 | 0 | 0 | 0 | 0 | 0 | **1** |
| Ireland | 0 | 0 | 0 | 0 | 0 | 0 | 0 | 0 | 1 | 0 | 0 | **1** |
| Israel | 0 | 0 | 0 | 0 | 1 | 0 | 0 | 0 | 0 | 0 | 0 | **1** |
| Italy | 0 | 24 | 0 | 4 | 3 | 6 | 1 | 1 | 0 | 0 | 0 | **39** |
| Japan | 0 | 1 | 43 | 57 | 22 | 4 | 8 | 0 | 0 | 0 | 0 | **135** |
| Mexico | 0 | 1 | 0 | 0 | 0 | 0 | 0 | 0 | 0 | 0 | 0 | **1** |
| Netherlands | 0 | 0 | 0 | 1 | 0 | 0 | 0 | 0 | 0 | 0 | 0 | **1** |
| New Zealand | 0 | 0 | 0 | 1 | 0 | 1 | 0 | 0 | 0 | 0 | 0 | **1** |
| Norway | 0 | 9 | 0 | 0 | 0 | 0 | 0 | 0 | 0 | 0 | 0 | **9** |
| Poland | 0 | 0 | 0 | 0 | 0 | 0 | 0 | 0 | 0 | 1 | 0 | **1** |
| Qatar | 0 | 0 | 0 | 0 | 0 | 0 | 0 | 1 | 0 | 0 | 0 | **1** |
| Romania | 24 | 2 | 41 | 42 | 0 | 0 | 0 | 171 | 78 | 41 | 0 | **399** |
| S. Korea | 0 | 0 | 1 | 1 | 0 | 0 | 0 | 0 | 0 | 0 | 0 | **2** |
| Saudi Arabia | 0 | 0 | 0 | 0 | 1 | 2 | 0 | 0 | 1 | 0 | 0 | **4** |
| Spain | 0 | 8 | 0 | 0 | 0 | 0 | 1 | 0 | 1 | 1 | 0 | **11** |
| Switzerland | 0 | 0 | 0 | 4 | 0 | 0 | 0 | 0 | 0 | 0 | 0 | **4** |
| Taiwan | 0 | 0 | 0 | 1 | 0 | 0 | 0 | 0 | 0 | 0 | 0 | **1** |
| Thailand | 0 | 0 | 0 | 0 | 1 | 0 | 0 | 0 | 0 | 0 | 0 | **1** |
| Turkey | 0 | 0 | 405 | 114 | 87 | 199 | 244 | 7 | 7 | 1 | 0 | **1064** |
| UK | 3 | 14 | 65 | 144 | 119 | 137 | 117 | 83 | 39 | 24 | 13 | **758** |
| USA | 3 | 39 | 10 | 20 | 14 | 2 | 3 | 6 | 5 | 4 | 0 | **106** |
| Uruguay | 0 | 0 | 0 | 0 | 0 | 0 | 0 | 1 | 0 | 0 | 0 | **1** |
| **Total** | **30** | **187** | **582** | **435** | **337** | **508** | **376** | **281** | **144** | **79** | **14** | **2973** |

**Figure S2.** Boxplots of coffee liking by **(a)** generation, **(b)** barista, **(c)** season, **(d)** weekday, **(e)** time of the day, **(f)** type of cup, **(g)** number of added ingredients, **(h)** number of coffees per day, **(i)** hours of sleep, and **(j)** wake-up hour. The central line in each box represents the median Liking score, while the box edges indicate the interquartile range (IQR: 25th to 75th percentile). The whiskers extend to 1.5 times the IQR, capturing most of the data distribution, while individual points beyond the whiskers represent potential outliers.

*Information-theory exploratory results*

Information gain (IG) results are presented Figures S3 and S4; see also Table S2 for the supporting descriptive data. The consistent high ranking of price across both metrics – IG and gain ratio – emphasizes its central importance in coffee preferences. Overall, the IG analysis identified that these 28 factors collectively explain nearly 31.17 % of the uncertainty in coffee preference. Given that the PPP-adjusted price explains most of uncertainty in measures of both IG and gain ratio, the main ***GLM(1)*** data analysis was thus performed on only those participants who reported the price paid for the consumed coffee, *N* = 2,522.

**Figure S3.** Percentage of uncertainty explained by predictor variables based on information gain analysis with coffee liking discretised into 6 categories (on a scale from 1 to 10). Values represent the percentage reduction in entropy (uncertainty) of coffee preference ratings, when each variable is known. Higher values indicate stronger predictors. With the highest IG (0.4054), price explains about 17% of the uncertainty in coffee liking scores. Wake-up hour, and country development become evident as the next most important explanatory factors.

**Figure S4.** Cumulative percentage of uncertainty explained by each group of variables categories. Values represent the sum of information gain percentages for all variables within each category, expressed as a percentage of total uncertainty (entropy) reduction. A higher percentage indicates that variables in that category collectively provide more information about coffee Liking. While individual variables may contain overlapping information, this cumulative view highlights those broad factors that impact the enjoyment of a cup of coffee most. **Demographics** include gender, generation, type of consumer, country development, and producer country; **Consumer habits** include the number of coffees consumed per day, waking hours, and hours of sleep; **Temporal context** includes season, time of the day, and weekday; **Intrinsic qualities** include Cocoa, Caramel, Cream, Decaffeinated, Ice, Milk, Sugar, No added ingredients, Total added ingredients; **Extrinsic qualities** include Lid on, Drinking alone, With company, Sitting, Standing, Walking, Type of cup, and PPP-adjusted price.

**Table S2.** Measures of coffee liking uncertainty explained.

| Rank^a^ | Variable | Information gain  [95% CI] ^b^ | %  Explained^c^ | Split info | Gain  ratio | Rank^d^ |
| --- | --- | --- | --- | --- | --- | --- |
| 1 | Price^e^ | .4054 [.375 .443] | 16.9200 | 1.8927 | 0.2142 | 1 |
| 2 | Waking hour^f^ | .0570 [.045 .074] | 2.3779 | 1.1232 | 0.0507 | 2 |
| 3 | Country development | .0425 [.032 .056] | 1.7737 | .9266 | 0.0459 | 3 |
| 4 | Number of coffees^f^ | .0202 [.015 .032] | 0.8441 | 1.1672 | 0.0173 | 4 |
| 5 | Hours of sleep^f^ | .0173 [.012 .029] | 0.7217 | 1.3050 | 0.0132 | 10 |
| 6 | Season | .0144 [.014 .027] | 0.6004 | 1.6281 | 0.0088 | 18 |
| 7 | Time of day | .0142 [.010 .025] | 0.5915 | 1.4532 | 0.0098 | 15 |
| 8 | Generation | .0137 [.011 .026] | 0.5703 | 1.6232 | 0.0084 | 20 |
| 9 | Type of cup | .0129 [.011 .026] | 0.5385 | 1.7103 | 0.0075 | 21 |
| 10 | Sugar | .0123 [.007 .020] | 0.5118 | .8259 | 0.0148 | 7 |
| 11 | Added ingredients^g^ | .0098 [.006 .020] | 0.4074 | 1.4407 | 0.0068 | 22 |
| 12 | Gender | .0097 [.007 .020] | 0.4042 | 1.0300 | 0.0094 | 17 |
| 13 | Producer country | .0091 [.005 .017] | 0.3810 | .7247 | 0.0126 | 11 |
| 14 | Ice | .0088 [.005 .016] | 0.3681 | .7074 | 0.0125 | 12 |
| 15 | Sitting | .0080 [.004 .016] | 0.3338 | .7129 | 0.0112 | 13 |
| 16 | Professional | .0067 [.004 .014] | 0.2871 | .4081 | 0.0169 | 5 |
| 17 | Weekday^f^ | .0057 [.005 .014] | 0.2399 | .8507 | 0.0068 | 23 |
| 18 | Cream | .0057 [.003 .013] | 0.2396 | .3975 | 0.0144 | 8 |
| 19 | Black coffee | .0043 [.002 .010] | 0.1780 | .9495 | 0.0045 | 24 |
| 20 | Caramel | .0040 [.002 .010] | 0.1690 | .4299 | 0.0094 | 16 |
| 21 | Standing | .0037 [.002 .010] | 0.1544 | .4208 | 0.0088 | 19 |
| 22 | Milk | .0037 [.002 .010] | 0.1544 | 1.0000 | 0.0037 | 25 |
| 23 | Walking | .0035 [.002 .009] | 0.1453 | .3506 | 0.0099 | 14 |
| 24 | Decaffeinated | .0034 [.002 .010] | 0.1429 | .2180 | 0.0157 | 6 |
| 25 | Drinking alone | .0033 [.001 .009] | 0.1385 | .9514 | 0.0035 | 26 |
| 26 | Cocoa | .0032 [.001 .009] | 0.1349 | .2385 | 0.0135 | 9 |
| 27 | With company | .0009 [.0004 .005] | 0.0370 | .9979 | 0.0009 | 28 |
| 28 | Lid on | .0008 [.0003 .005] | 0.0323 | .5231 | 0.0015 | 27 |

*Notes:* The base entropy for liking discretised on 6 categories is 2.3958 bits.

^a^ Rank based on the calculated information gain.

^b^ Information gain is expressed in bits and indicates how much uncertainty about coffee preference is reduced by knowing the value of the predictor variable. 95% CIs were obtained through bootstrap resampling; *n* = 1,000.

^c^ Percentage Explained = (Information Gain / Base Entropy) × 100. It converts the raw information gain (expressed in bits) to a percentage of the total entropy.

^d^ Rank based on gain ratio.

^e^ PPP-adjusted price is discretised in quartiles.

^f^ Variables that are categorised as detailed in Table 1 (Summary statistics).

^g^ Added ingredients are categorized as Black coffee (no ingredients), Low (1-2 added ingredients), and High (more than 3 added ingredients).


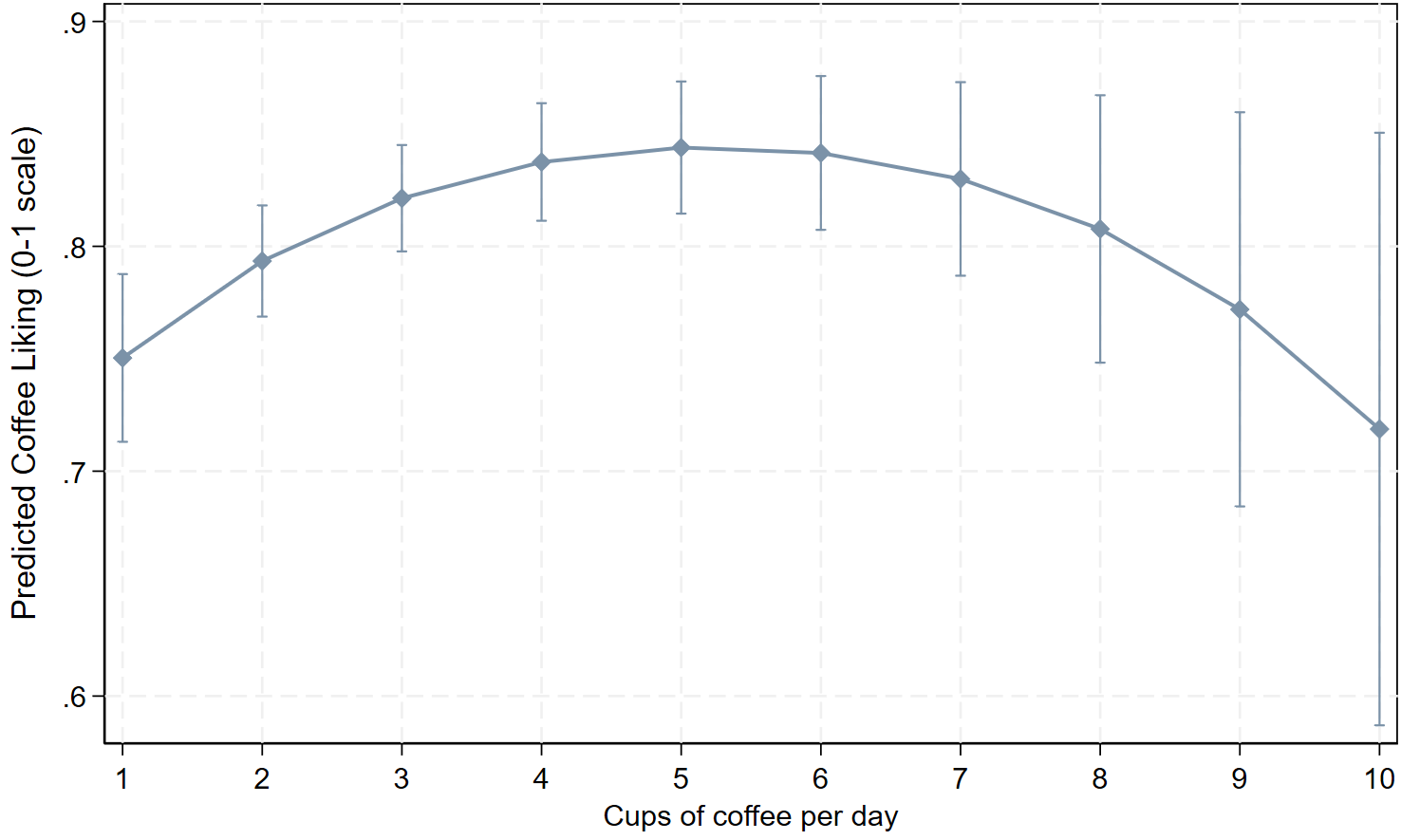


**Figure S5.** Predicted coffee liking (0-1 scale) by number of coffees per day from the Baseline fractional response (FR) model with robust clustered errors.

**Table S3.** Hierarchical fractional logit (FR) model: logit coefficients.

|  | Baseline FR | | Temporal FR | | Demographic FR | |
| --- | --- | --- | --- | --- | --- | --- |
|  | β^a^ | β^b^ | β^a^ | β^b^ | β^a^ | β^b^ |
| Cream | -0.206^*^  (0.101) | -0.200^**^  (0.077) | -0.047  (0.218) | -0.027  (0.192) | -0.041  (0.219) | -0.020  (0.210) |
| Milk | 0.139  (0.085) | 0.137  (0.075) | 0.144  (0.086) | 0.143  (0.078) | 0.817^*^  (0.337) | 0.813^***^  (0.196) |
| Sugar | -0.193^**^  (0.071) | -0.191^**^  (0.069) | -0.191^**^  (0.071) | -0.187^**^  (0.071) | -0.209^**^  (0.073) | -0.205^**^  (0.068) |
| Black coffee | -0.097  (0.093) | -0.098  (0.122) | -0.332^*^  (0.150) | -0.338  (0.203) | -0.321^*^  (0.149) | -0.328  (0.198) |
| Lid on | 0.216^*^  (0.098) | 0.214^*^  (0.096) | 0.652^*^  (0.272) | 0.708  (0.398) | 0.651^*^  (0.284) | 0.693  (0.394) |
| Drinking alone | 0.122^*^  (0.056)^***^ | 0.128  (0.083) | -0.166  (0.221) | -0.139  (0.165) | -0.180  (0.223) | -0.171  (0.173) |
| PPP-adj. price | 0.036^*^  (0.010) | 0.035^***^  (0.006) | 0.034^***^  (0.010) | 0.033^***^  (0.005) | 0.035^***^  (0.010) | 0.034^***^  (0.005) |
| Premium cup | 0.157^*^  (0.079) | 0.167  (0.090) | 0.301^*^  (0.122) | 0.331^***^  (0.072) | 0.302^*^  (0.122) | 0.332^***^  (0.079) |
| Number of coffee cups | 0.348^***^  (0.054) | 0.349^***^  (0.082) | 0.349^***^  (0.054) | 0.350^***^  (0.077) | 0.347^***^  (0.054) | 0.348^***^  (0.076) |
| Number of coffee cups squared | -0.033^***^  (0.007) | -0.033^***^  (0.009) | -0.034^***^  (0.007) | -0.034^***^  (0.009) | -0.034^***^  (0.007) | -0.034^***^  (0.008) |
| Waking hour | -0.018  (0.022) | -0.018  (0.028) | -0.019  (0.022) | -0.018  (0.027) | -0.275^*^  (0.115) | -0.291^***^  (0.081) |
| *Season* |  |  |  |  |  |  |
| Summer | -0.290^*^  (0.121) | -0.233  (0.164) | -0.285  (0.177) | -0.214  (0.256) | -0.268  (0.179) | -0.221  (0.259) |
| Autumn | -0.473^***^  (0.124) | -0.425^*^  (0.182) | -0.584^***^  (0.179) | -0.519  (0.330) | -0.572^**^  (0.181) | -0.533  (0.334) |
| Winter | -0.090  (0.136) | -0.061  (0.255) | -0.125  (0.196) | -0.082  (0.313) | -0.104  (0.199) | -0.081  (0.318) |
| Weekday | 0.012  (0.013) | 0.012  (0.020) | -0.024  (0.024) | -0.026^***^  (0.006) | -0.025  (0.023) | -0.026^***^  (0.006) |
| *Time of day* |  |  |  |  |  |  |
| Noon/Afternoon | -0.218^***^  (0.059) | -0.228^**^  (0.084) | -0.066  (0.129) | -0.061  (0.116) | -0.079  (0.130) | -0.074  (0.112) |
| Evening | -0.319^***^  (0.059) | -0.330^**^  (0.128) | -0.072  (0.164) | -0.057  (0.097) | -0.078  (0.164) | -0.063  (0.098) |
| *Generation* |  |  |  |  |  |  |
| GenX | 0.304  (0.173) | 0.333  (0.186) | 0.305  (0.173) | 0.333^*^  (0.155) | -0.757  (0.865) | -0.841  (0.660) |
| Millennials | 0.318  (0.164) | 0.346^*^  (0.155) | 0.322  (0.164) | 0.350^**^  (0.136) | -0.784  (0.808) | -0.882  (0.537) |
| Gen Z | 0.308  (0.167) | 0.343  (0.197) | 0.310  (0.167) | 0.346^*^  (0.176) | -1.369  (0.790) | -1.466^*^  (0.567) |
| *Season* × *Lid on* |  |  |  |  |  |  |
| Summer × Lid on |  |  | -0.511  (0.287) | -0.574  (0.466) | -0.492  (0.299) | -0.540  (0.468) |
| Autumn × Lid on |  |  | -0.266  (0.306) | -0.313  (0.448) | -0.260  (0.318) | -0.292  (0.433) |
| Winter × Lid on |  |  | -0.823^*^  (0.341) | -0.895^*^  (0.396) | -0.811^*^  (0.349) | -0.866^*^  (0.392) |
| *Season* × *Drinking alone* |  |  |  |  |  |  |
| Summer × Drinking alone |  |  | 0.163  (0.236) | 0.142  (0.140) | 0.169  (0.237) | 0.166  (0.149) |
| Autumn × Drinking alone |  |  | 0.432  (0.242) | 0.401  (0.262) | 0.450  (0.244) | 0.437  (0.266) |
| Winter × Drinking alone |  |  | 0.354  (0.269) | 0.334^*^  (0.146) | 0.358  (0.271) | 0.349^*^  (0.142) |
|  |  |  |  |  |  |  |
| *Weekday* × *Black coffee* |  |  | 0.055  (0.028) | 0.057^*^  (0.024) | 0.054  (0.028) | 0.055^*^  (0.023) |
|  |  |  |  |  |  |  |
| *Time of day* × *Cream* |  |  |  |  |  |  |
| Noon × Cream |  |  | 0.049  (0.254) | 0.036  (0.203) | 0.048  (0.255) | 0.035  (0.210) |
| Evening × Cream |  |  | -0.469  (0.284) | -0.488^*^  (0.247) | -0.473  (0.285) | -0.493^*^  (0.253) |
| *Time of day* × *Premium cup* |  |  |  |  |  |  |
| Noon × Premium cup |  |  | -0.219  (0.143) | -0.239^**^  (0.078) | -0.206  (0.144) | -0.224^**^  (0.073) |
| Evening × Premium cup |  |  | -0.222  (0.193) | -0.256  (0.153) | -0.217  (0.192) | -0.251  (0.137) |
| *Generation* × *Waking hour* |  |  |  |  |  |  |
| Gen X × Waking hour |  |  |  |  | 0.214  (0.130) | 0.228^**^  (0.083) |
| Millennials × Waking hour |  |  |  |  | 0.222  (0.122) | 0.238^**^  (0.077) |
| Gen Z × Waking hour |  |  |  |  | 0.291^*^  (0.119) | 0.308^***^  (0.082) |
| *Generation* × *Milk* |  |  |  |  |  |  |
| Gen X × Milk |  |  |  |  | -0.763^*^  (0.365) | -0.768^***^  (0.224) |
| Millennials × Milk |  |  |  |  | -0.740^*^  (0.345) | -0.733^***^  (0.195) |
| Gen Z ×Milk |  |  |  |  | -0.638  (0.344) | -0.635^**^  (0.271) |
| Constant | 0.701^**^  (0.273) | 0.619  (0.340) | 0.799^*^  (0.328) | 0.694^*^  (0.326) | 2.178^**^  (0.789) | 2.226^***^  (0.692) |
| *N* | 2,466 | 2,411 | 2,466 | 2,411 | 2,466 | 2,411 |
| *AIC* | 2488.508 | 2415.131 | 2503.691 | 2408.109 | 2512.476 | 2404.96 |
| *Pseudo R2* | 0.0212 | 0.0210 | 0.0239 | 0.0239 | 0.0252 | 0.0252 |
| Joint Significance (Wald test *χ2*) |  |  | 98.87^***^ | 391.26^***^ | 114.41^***^ | 155.24^***^ |

*Notes:* ^a^Robust standard errors are in parenthesis; ^b^Robust standard errors clustered by recruitment country. To ensure robust variance estimation, the analysis is restricted to countries with a minimum of 30 observations (*n* = 9 countries). Premium cup includes ceramic and glass cups. ^*^*p*<.05, ^**^*p*<.01, ^***^*p*<.001.

**Table S4.** Hierarchical fractional response (FR) model: Average marginal effects.

|  | **Baseline FR** | | **Temporal FR** | | **Demographic FR** | |
| --- | --- | --- | --- | --- | --- | --- |
|  | *dy/dx ^a^* | *dy/dx ^b^* | *dy/dx ^a^* | *dy/dx ^b^* | *dy/dx ^a^* | *dy/dx ^b^* |
| Cream | -0.034  (0.018) | -0.033^**^  (0.013) | -0.017  (0.017) | -0.016  (0.015) | -0.017  (0.017) | -0.015  (0.016) |
| Milk | 0.022  (0.014) | 0.022  (0.012) | 0.023  (0.014) | 0.023  (0.012) | 0.024  (0.014) | 0.024  (0.012) |
| Sugar | -0.032^**^  (0.012) | -0.031^**^  (0.012) | -0.031^**^  (0.012) | -0.031^*^  (0.012) | -0.034^**^  (0.012) | -0.033^**^  (0.012) |
| Black coffee | -0.015  (0.015) | -0.015  (0.019) | -0.019  (0.014) | -0.019  (0.019) | -0.018  (0.015) | -0.018  (0.018) |
| Lid on | 0.033^*^  (0.014) | 0.033^*^  (0.014) | 0.031^*^  (0.014) | 0.031^*^  (0.012) | 0.033^*^  (0.014) | 0.032^**^  (0.012) |
| Drinking alone | 0.019^*^  (0.009) | 0.020  (0.014) | 0.017  (0.009) | 0.018  (0.013) | 0.016  (0.009) | 0.017  (0.013) |
| PPP-adj. price | 0.006^***^  (0.002) | 0.006^***^  (0.001) | 0.005^***^  (0.002) | 0.005^***^  (0.001) | 0.006^***^  (0.002) | 0.005^***^  (0.001) |
| Premium cup | 0.026  (0.013) | 0.027  (0.014) | 0.026^*^  (0.013) | 0.028^**^  (0.010) | 0.027^*^  (0.013) | 0.030^**^  (0.010) |
| Number of coffee cups | 0.031^***^  (0.005) | 0.031^***^  (0.008) | 0.030^***^  (0.004) | 0.030^***^  (0.007) | 0.030^***^  (0.005) | 0.030^***^  (0.007) |
| Waking hour | -0.003  (0.003) | -0.003  (0.004) | -0.003  (0.003) | -0.003  (0.004) | -0.005  (0.003) | -0.005  (0.003) |
| *Season* |  |  |  |  |  |  |
| Summer | -0.042^**^  (0.016) | -0.034  (0.022) | -0.041^*^  (0.017) | -0.033  (0.024) | -0.038^*^  (0.017) | -0.032  (0.023) |
| Autumn | -0.072^***^  (0.017) | -0.066^**^  (0.025) | -0.070^***^  (0.018) | -0.064^*^  (0.031) | -0.068^***^  (0.018) | -0.064^*^  (0.031) |
| Winter | -0.012  (0.018) | -0.008  (0.035) | -0.014  (0.019) | -0.010  (0.037) | -0.011  (0.019) | -0.009  (0.037) |
| Weekday | 0.002  (0.002) | 0.002  (0.003) | 0.002  (0.002) | 0.002  (0.003) | 0.002  (0.002) | 0.002  (0.003) |
| *Time of day* |  |  |  |  |  |  |
| Noon/Afternoon | -0.034^***^  (0.009) | -0.035^**^  (0.014) | -0.034^***^  (0.009) | -0.036^*^  (0.014) | -0.035^***^  (0.009) | -0.036^**^  (0.014) |
| Evening | -0.051^***^  (0.015) | -0.053^*^  (0.021) | -0.044^**^  (0.015) | -0.046^*^  (0.021) | -0.045^**^  (0.015) | -0.047^*^  (0.020) |
| *Generation* |  |  |  |  |  |  |
| Gen X | 0.052  (0.031) | 0.058  (0.034) | 0.052  (0.031) | 0.058^*^  (0.029) | 0.085^*^  (0.039) | 0.090^***^  (0.024) |
| Millennials | 0.055  (0.030) | 0.060^*^  (0.029) | 0.055  (0.030) | 0.061^*^  (0.026) | 0.093^*^  (0.037) | 0.098^***^  (0.029) |
| Gen Z | 0.053  (0.031) | 0.060  (0.035) | 0.053  (0.031) | 0.060  (0.031) | 0.091^*^  (0.037) | 0.097^**^  (0.035) |
| *Season* × *Lid on* |  |  |  |  |  |  |
| Summer × Without Lid on |  |  | -0.034  (0.019) | -0.024  (0.030) | -0.031  (0.019) | -0.024  (0.030) |
| Summer × Lid on |  |  | -0.085^***^  (0.026) | -0.085^*^  (0.036) | -0.080^**^  (0.027) | -0.081^*^  (0.036) |
| Autumn × Without Lid on |  |  | -0.068^***^  (0.019) | -0.061  (0.037) | -0.066^***^  (0.020) | -0.061  (0.037) |
| Autumn × Lid on |  |  | -0.079^**^  (0.030) | -0.079^**^  (0.030) | -0.077^*^  (0.031) | -0.077^**^  (0.027) |
| Winter × Without Lid on |  |  | -0.001  (0.021) | 0.005  (0.043) | 0.003  (0.021) | 0.006  (0.043) |
| Winter × Lid on |  |  | -0.098^**^  (0.037) | -0.104^***^  (0.015) | -0.094^*^  (0.038) | -0.099^***^  (0.015) |
| *Season* × *Drinking alone* |  |  |  |  |  |  |
| Summer × Drinking with company |  |  | -0.048^*^  (0.022) | -0.040  (0.027) | -0.046^*^  (0.023) | -0.040  (0.028) |
| Summer × Drinking alone |  |  | -0.027  (0.023) | -0.020  (0.019) | -0.023  (0.024) | -0.017  (0.019) |
| Autumn × Drinking with company |  |  | -0.094^***^  (0.023) | -0.086^*^  (0.043) | -0.093^***^  (0.024) | -0.088^*^  (0.043) |
| Autumn × Drinking alone |  |  | -0.027  (0.025) | -0.023  (0.019) | -0.022  (0.025) | -0.019  (0.018) |
| Winter × Drinking with company |  |  | -0.031  (0.025) | -0.027  (0.038) | -0.028  (0.025) | -0.026  (0.038) |
| Winter × Drinking alone |  |  | 0.017  (0.027) | 0.020  (0.040) | 0.021  (0.027) | 0.023  (0.039) |
| Weekday × Added ingredients |  |  | -0.004  (0.004) | -0.004^***^  (0.001) | -0.004  (0.004) | -0.004^***^  (0.001) |
| Weekday × Black coffee |  |  | 0.005  (0.003) | 0.005  (0.004) | 0.005  (0.003) | 0.005  (0.004) |
| *Time of day* × *Cream* |  |  |  |  |  |  |
| Noon × No cream |  |  | -0.035^***^  (0.009) | -0.036^**^  (0.013) | -0.035^***^  (0.009) | -0.037^**^  (0.013) |
| Noon × Cream |  |  | -0.028  (0.038) | -0.031  (0.038) | -0.028  (0.038) | -0.031  (0.038) |
| Evening × No cream |  |  | -0.036^*^  (0.016) | -0.038  (0.020) | -0.037^*^  (0.016) | -0.038^*^  (0.019) |
| Evening × Cream |  |  | -0.127^**^  (0.047) | -0.132^*^  (0.052) | -0.128^**^  (0.047) | -0.133^**^  (0.051) |
| *Time of day* × *Premium cup* |  |  |  |  |  |  |
| Noon × No premium cup |  |  | -0.010  (0.021) | -0.010  (0.022) | -0.013  (0.021) | -0.012  (0.021) |
| Noon × Premium cup |  |  | -0.042^***^  (0.010) | -0.045^***^  (0.013) | -0.042^***^  (0.010) | -0.044^***^  (0.012) |
| Evening × No premium cup |  |  | -0.020  (0.027) | -0.018  (0.017) | -0.021  (0.027) | -0.019  (0.018) |
| Evening × Premium cup |  |  | -0.052^**^  (0.018) | -0.056^*^  (0.026) | -0.053^**^  (0.018) | -0.056^*^  (0.024) |
| *Generation* × *Waking hour* |  |  |  |  |  |  |
| Boomers × Waking hour |  |  |  |  | -0.053^*^  (0.023) | -0.057^***^  (0.016) |
| Gen X × Waking hour |  |  |  |  | -0.010  (0.010) | -0.010  (0.007) |
| Millennials × Waking hour |  |  |  |  | -0.008  (0.006) | -0.009^***^  (0.002) |
| Gen Z × Waking hour |  |  |  |  | 0.002  (0.005) | 0.003  (0.005) |
| *Generation* ×*Milk* |  |  |  |  |  |  |
| Gen X × Without Milk |  |  |  |  | 0.168^**^  (0.058) | 0.173^***^  (0.040) |
| Gen X × Milk |  |  |  |  | 0.019  (0.044) | 0.022  (0.022) |
| Millennials × Without Milk |  |  |  |  | 0.174^**^  (0.057) | 0.179^***^  (0.036) |
| Millennials × Milk |  |  |  |  | 0.028  (0.041) | 0.033  (0.032) |
| Gen Z × Without Milk |  |  |  |  | 0.163^**^  (0.057) | 0.169^***^  (0.038) |
| Gen Z × Milk |  |  |  |  | 0.034  (0.041) | 0.039  (0.044) |
| *N* | 2,466 | 2,411 | 2,466 | 2,411 | 2,466 | 2,411 |

*Notes:* ^a^FR model with robust SE; ^b^FR model with robust SE clustered by recruitment country. These estimates are derived from the FR model in which the dependent variable, coffee Liking, was rescaled to the [0,1] interval. Thus, a margin measures the change in the expected proportion of the maximum score for a one-unit change in a covariate (with 1 as the highest possible liking on the original scale). The margins are computed by holding all other covariates in the model at their observed values. Delta-method was used to calculate SEs. ^*^*p*<.05, ^**^*p*<.01, ^***^*p*<.001.

**Table S5.** Marginal effects for ZOIB model.

|  | Beta equation | | One-Inflation equation | |
| --- | --- | --- | --- | --- |
|  | *dy/dx* | *SE* | *dy/dx* | *SE* |
| Cream | -0.023 | 0.016 | -0.028 | 0.036 |
| Milk | 0.039^**^ | 0.013 | -0.056^*^ | 0.027 |
| Sugar | -0.011 | 0.011 | -0.056^*^ | 0.025 |
| Black coffee | -0.032^*^ | 0.014 | 0.048 | 0.028 |
| Lid on | 0.022 | 0.014 | 0.038 | 0.034 |
| Drinking alone | 0.000 | 0.008 | 0.037 | 0.020 |
| PPP-adj. price | 0.002 | 0.001 | 0.013^***^ | 0.003 |
| Premium cup | 0.023 | 0.012 | 0.026 | 0.026 |
| Number of coffee cups | 0.008^*^ | 0.004 | 0.062^***^ | 0.008 |
| Waking hour | -0.001 | 0.003 | -0.004 | 0.007 |
| *Season* |  |  |  |  |
| Summer | -0.041^**^ | 0.014 | -0.011 | 0.047 |
| Autumn | -0.044^**^ | 0.015 | -0.070 | 0.048 |
| Winter | -0.019 | 0.016 | 0.028 | 0.052 |
| Weekday | 0.005^**^ | 0.002 | -0.006 | 0.005 |
| *Time of day* |  |  |  |  |
| Noon/Afternoon | -0.021^*^ | 0.008 | -0.033 | 0.021 |
| Evening | -0.033^*^ | 0.013 | -0.009 | 0.031 |
| *Generation* |  |  |  |  |
| Gen X | 0.044 | 0.027 | -0.007 | 0.055 |
| Millennials | 0.030 | 0.027 | 0.027 | 0.052 |
| Gen Z | 0.015 | 0.027 | 0.046 | 0.053 |
| *N* | 2,466 |  | 2,466 |  |

*Notes:* 1. Beta coefficients represent marginal effects on the conditional mean of rescaled Liking for observations where 0 < Liking < 1. One-inflation coefficients represent marginal effects on the probability that Liking = 1 (maximum value). ^*^*p*<.05, ^**^*p*<.01, ^***^*p*<.001.

**Table S6.** Linear mixed model (MLM) estimates.

|  | **Baseline MLM** | | **Temporal MLM** | | **Demographic MLM** | |
| --- | --- | --- | --- | --- | --- | --- |
|  | *β* | *SE* | *β* | *SE* | *β* | *SE* |
| Cream | -0.021 | 0.014 | 0.012 | 0.028 | 0.014 | 0.031 |
| Milk | 0.027 | 0.017 | 0.026 | 0.018 | 0.153^***^ | 0.029 |
| Sugar | -0.043^***^ | 0.010 | -0.043^***^ | 0.011 | -0.044^***^ | 0.011 |
| Black coffee | -0.020 | 0.015 | -0.055 | 0.031 | -0.054 | 0.031 |
| Lid on | 0.023 | 0.019 | 0.106 | 0.055 | 0.101 | 0.055 |
| Drinking alone | 0.022 | 0.015 | 0.003 | 0.013 | -0.002 | 0.014 |
| PPP-adj. price | 0.007^***^ | 0.001 | 0.007^***^ | 0.001 | 0.007^***^ | 0.001 |
| Premium cup | 0.018 | 0.011 | 0.041^***^ | 0.010 | 0.041^***^ | 0.010 |
| Number of coffee cups | 0.060^***^ | 0.015 | 0.060^***^ | 0.015 | 0.060^***^ | 0.014 |
| Number of coffee cups squared | -0.006^***^ | 0.002 | -0.006^***^ | 0.002 | -0.006^***^ | 0.002 |
| Waking hour | -0.002 | 0.006 | -0.002 | 0.006 | -0.046^***^ | 0.013 |
| *Season* |  |  |  |  |  |  |
| Summer | -0.012 | 0.013 | 0.001 | 0.014 | -0.001 | 0.015 |
| Autumn | -0.049^**^ | 0.017 | -0.052 | 0.036 | -0.055 | 0.036 |
| Winter | -0.030 | 0.025 | -0.019 | 0.026 | -0.021 | 0.027 |
| Weekday | -0.001 | 0.003 | -0.006^***^ | 0.001 | -0.006^***^ | 0.001 |
| *Time of day* |  |  |  |  |  |  |
| Noon/Afternoon | -0.028^*^ | 0.014 | -0.004 | 0.015 | -0.006 | 0.014 |
| Evening | -0.035 | 0.021 | 0.012 | 0.009 | 0.011 | 0.009 |
| *Generation* |  |  |  |  |  |  |
| GenX | 0.049 | 0.035 | 0.050 | 0.031 | -0.118 | 0.081 |
| Millennials | 0.050 | 0.030 | 0.053 | 0.028 | -0.137 | 0.084 |
| GenZ | 0.046 | 0.033 | 0.050 | 0.031 | -0.221^*^ | 0.089 |
| *Season* × *Lid on* |  |  |  |  |  |  |
| Summer × Lid on |  |  | -0.091 | 0.064 | -0.084 | 0.065 |
| Autumn × Lid on |  |  | -0.065 | 0.054 | -0.059 | 0.054 |
| Winter × Lid on |  |  | -0.142^**^ | 0.053 | -0.136^*^ | 0.054 |
| *Season* × *Drinking alone* |  |  |  |  |  |  |
| Summer × Drinking alone |  |  | 0.001 | 0.015 | 0.004 | 0.015 |
| Autumn × Drinking alone |  |  | 0.042 | 0.042 | 0.048 | 0.042 |
| Winter × Drinking alone |  |  | 0.019 | 0.025 | 0.022 | 0.024 |
| *Weekday* × *Black coffee* |  |  | 0.008 | 0.005 | 0.008 | 0.004 |
| *Time of day* × *Cream* |  |  |  |  |  |  |
| Noon × Cream |  |  | 0.010 | 0.033 | 0.009 | 0.034 |
| Evening × Cream |  |  | -0.108 | 0.044 | -0.109^*^ | 0.045 |
| *Time of day* × *Premium cup* |  |  |  |  |  |  |
| Noon × Premium cup |  |  | -0.034^***^ | 0.011 | -0.032^***^ | 0.010 |
| Evening × Premium cup |  |  | -0.038 | 0.025 | -0.038 | 0.022 |
| *Generation* × *Waking hour* |  |  |  |  |  |  |
| Gen X × Waking hour |  |  |  |  | 0.036^***^ | 0.010 |
| Millennials × Waking hour |  |  |  |  | 0.039^**^ | 0.013 |
| Gen Z × Waking hour |  |  |  |  | 0.049^***^ | 0.014 |
| *Generation* × *Milk* |  |  |  |  |  |  |
| Gen X × Milk |  |  |  |  | -0.135^***^ | 0.038 |
| Millennials × Milk |  |  |  |  | -0.132^***^ | 0.033 |
| Gen Z × Milk |  |  |  |  | -0.126^***^ | 0.038 |
| Constant | 0.677^***^ | 0.085 | 0.673^***^ | 0.068 | 0.902^***^ | 0.095 |
| $\sigma_{u}^{2}$ | 0.002 | 0.001 | 0.002 | 0.001 | 0.002 | 0.001 |
| $\sigma_{e}^{2}$ | 0.044 | 0.010 | 0.044 | 0.010 | 0.044 | 0.010 |
| *N* | 2,411 | | 2,411 | | 2,411 | |
| *AIC* | -600.1012 | | -604.6082 | | -604.0934 | |
| Joint Significance (Wald test *χ2*) |  | | 329.96^***^ | | 157.38^***^ | |

*Note:* Estimates are derived from a linear mixed model with random intercepts for recruitment country. Robust standard errors (*SE*). The sample is restricted to the primary multi-country subset (*n* = 9 countries). Premium cup includes ceramic and glass cups. ^*^*p*<.05, ^**^*p*<.01, ^***^*p*<.001.

**Table S7.** Linear mixed model (MLM): Average marginal effects.

|  | **Baseline FR** | | **Temporal FR** | | **Demographic FR** | |
| --- | --- | --- | --- | --- | --- | --- |
|  | *dy/dx* | *SE* | *dy/dx* | *SE* | *dy/dx* | *SE* |
| Cream | -0.021 | 0.014 | -0.000 | 0.011 | 0.001 | 0.012 |
| Milk | 0.027 | 0.017 | 0.026 | 0.018 | 0.027 | 0.018 |
| Sugar | -0.043^***^ | 0.010 | -0.043^***^ | 0.011 | -0.044^***^ | 0.011 |
| Black coffee | -0.020 | 0.015 | -0.024 | 0.015 | -0.024 | 0.015 |
| Lid on | 0.023 | 0.019 | 0.020 | 0.018 | 0.022 | 0.018 |
| Drinking alone | 0.022 | 0.015 | 0.019 | 0.014 | 0.019 | 0.014 |
| PPP-adj. price | 0.007^***^ | 0.001 | 0.007^***^ | 0.001 | 0.007^***^ | 0.001 |
| Premium cup | 0.018 | 0.011 | 0.020^**^ | 0.008 | 0.021^**^ | 0.008 |
| Number of coffee cups | 0.031^***^ | 0.008 | 0.031^***^ | 0.007 | 0.030^***^ | 0.007 |
| Waking hour | -0.002 | 0.006 | -0.002 | 0.006 | -0.004 | 0.004 |
| *Season* |  |  |  |  |  |  |
| Summer | -0.012 | 0.013 | -0.010 | 0.008 | -0.010 | 0.008 |
| Autumn | -0.049^**^ | 0.017 | -0.046^*^ | 0.018 | -0.046^*^ | 0.018 |
| Winter | -0.030 | 0.025 | -0.031 | 0.024 | -0.030 | 0.024 |
| Weekday | -0.001 | 0.003 | -0.001 | 0.003 | -0.001 | 0.003 |
| *Time of day* |  |  |  |  |  |  |
| Noon/Afternoon | -0.028^*^ | 0.014 | -0.028^*^ | 0.014 | -0.029^*^ | 0.014 |
| Evening | -0.035 | 0.021 | -0.027 | 0.019 | -0.027 | 0.019 |
| *Generation* |  |  |  |  |  |  |
| Gen X | 0.049 | 0.035 | 0.050 | 0.031 | 0.074^**^ | 0.025 |
| Millennials | 0.050 | 0.030 | 0.053 | 0.028 | 0.083^**^ | 0.030 |
| Gen Z | 0.046 | 0.033 | 0.050 | 0.031 | 0.079^*^ | 0.033 |
| *Season* × *Lid on* |  |  |  |  |  |  |
| Summer × Without Lid on |  |  | 0.002 | 0.012 | 0.001 | 0.013 |
| Summer × Lid on |  |  | -0.089 | 0.055 | -0.083 | 0.056 |
| Autumn × Without Lid on |  |  | -0.038 | 0.023 | -0.038 | 0.023 |
| Autumn × Lid on |  |  | -0.103^**^ | 0.040 | -0.098^**^ | 0.039 |
| Winter × Without Lid on |  |  | -0.012 | 0.029 | -0.013 | 0.029 |
| Winter × Lid on |  |  | -0.154^***^ | 0.035 | -0.149^*^ | 0.036 |
| *Season* × *Drinking alone* |  |  |  |  |  |  |
| Summer × Drinking with company |  |  | -0.010 | 0.008 | -0.011 | 0.009 |
| Summer × Drinking alone |  |  | -0.010 | 0.015 | -0.007 | 0.014 |
| Autumn × Drinking with company |  |  | -0.061 | 0.032 | -0.063^*^ | 0.032 |
| Autumn × Drinking alone |  |  | -0.019 | 0.015 | -0.015 | 0.014 |
| Winter × Drinking with company |  |  | -0.037 | 0.020 | -0.038 | 0.022 |
| Winter × Drinking alone |  |  | -0.019 | 0.036 | -0.016 | 0.035 |
| Weekday × Added ingredients |  |  | -0.006^***^ | 0.001 | -0.006^***^ | 0.001 |
| Weekday × Black coffee |  |  | 0.002 | 0.004 | 0.002 | 0.004 |
| *Time of day* × *Cream* |  |  |  |  |  |  |
| Noon × No cream |  |  | -0.029^*^ | 0.012 | -0.029^*^ | 0.012 |
| Noon × Cream |  |  | -0.020 | 0.042 | -0.020 | 0.043 |
| Evening × No cream |  |  | -0.017 | 0.017 | -0.017 | 0.017 |
| Evening × Cream |  |  | -0.126^*^ | 0.056 | -0.126^*^ | 0.056 |
| *Time of day* × *Premium cup* |  |  |  |  |  |  |
| Noon × No premium cup |  |  | -0.003 | 0.017 | -0.005 | 0.016 |
| Noon × Premium cup |  |  | -0.037^**^ | 0.014 | -0.037^**^ | 0.014 |
| Evening × No premium cup |  |  | 0.002 | 0.009 | 0.002 | 0.010 |
| Evening × Premium cup |  |  | -0.036 | 0.025 | -0.036 | 0.024 |
| *Generation* × *Waking hour* |  |  |  |  |  |  |
| Boomers × Waking hour |  |  |  |  | -0.046^***^ | 0.013 |
| Gen X × Waking hour |  |  |  |  | -0.010 | 0.007 |
| Millennials × Waking hour |  |  |  |  | -0.007^***^ | 0.002 |
| Gen Z × Waking hour |  |  |  |  | 0.003 | 0.007 |
| *Generation* ×*Milk* |  |  |  |  |  |  |
| Gen X × Without Milk |  |  |  |  | 0.145^***^ | 0.042 |
| Gen X × Milk |  |  |  |  | 0.010 | 0.019 |
| Millennials × Without Milk |  |  |  |  | 0.152^***^ | 0.040 |
| Millennials × Milk |  |  |  |  | 0.021 | 0.028 |
| Gen Z × Without Milk |  |  |  |  | 0.146^***^ | 0.039 |
| Gen Z × Milk |  |  |  |  | 0.020 | 0.037 |
| *N* | 2,411 |  | 2,411 |  | 2,411 |  |

*Notes:* These estimates are derived from the LMM model in which the dependent variable, coffee Liking, was rescaled to the [0,1] interval. Thus, a margin measures the change in the expected proportion of the maximum score for a one-unit change in a covariate (with 1 as the highest possible liking on the original scale). The margins are computed by holding all other covariates in the model at their observed values. Delta-method was used to calculate SEs. ^*^*p*<.05, ^**^*p*<.01, ^***^*p*<.001.

**Table S8.** Fractional response (FR) regression for the sample without Turkey: Average marginal effects.

|  | **Baseline FR** | | **Temporal FR** | | **Demographic FR** | |
| --- | --- | --- | --- | --- | --- | --- |
|  | *dy/dx* | *SE* | *dy/dx* | *SE* | *dy/dx* | *SE* |
| Cream | -0.007 | 0.020 | -0.001 | 0.019 | -0.000 | 0.022 |
| Milk | 0.019 | 0.014 | 0.021 | 0.014 | 0.023 | 0.015 |
| Sugar | -0.030^*^ | 0.014 | -0.031^*^ | 0.013 | -0.036^**^ | 0.012 |
| Black coffee | 0.003 | 0.014 | 0.002 | 0.015 | 0.003 | 0.015 |
| Lid on | 0.013 | 0.015 | 0.008 | 0.011 | 0.009 | 0.011 |
| Drinking alone | 0.005 | 0.012 | 0.005 | 0.011 | 0.003 | 0.011 |
| PPP-adj. price | 0.006^***^ | 0.001 | 0.006^***^ | 0.001 | 0.006^***^ | 0.001 |
| Premium cup | 0.037^*^ | 0.015 | 0.033^*^ | 0.015 | 0.035^*^ | 0.016 |
| Number of coffee cups | 0.022^***^ | 0.006 | 0.023^***^ | 0.006 | 0.022^***^ | 0.006 |
| Waking hour | -0.005 | 0.004 | -0.005 | 0.004 | -0.007^*^ | 0.003 |
| *Season* |  |  |  |  |  |  |
| Summer | -0.039^*^ | 0.019 | -0.032 | 0.020 | -0.030 | 0.021 |
| Autumn | -0.038^*^ | 0.018 | -0.031 | 0.025 | -0.030 | 0.026 |
| Winter | -0.008 | 0.029 | -0.005 | 0.033 | -0.003 | 0.034 |
| Weekday | -0.003 | 0.003 | -0.002 | 0.003 | -0.003 | 0.003 |
| *Time of day* |  |  |  |  |  |  |
| Noon/Afternoon | -0.021 | 0.017 | -0.022 | 0.013 | -0.022 | 0.012 |
| Evening | 0.009 | 0.018 | 0.023 | 0.018 | 0.022 | 0.018 |
| *Generation* |  |  |  |  |  |  |
| Gen X | 0.059 | 0.035 | 0.059^*^ | 0.029 | 0.072^**^ | 0.023 |
| Millennials | 0.061^*^ | 0.030 | 0.061^*^ | 0.026 | 0.077^**^ | 0.027* |
| Gen Z | 0.080^**^ | 0.031 | 0.079^**^ | 0.028 | 0.093^**^ | 0.031 |
| *Season* × *Lid on* |  |  |  |  |  |  |
| Summer × Without Lid on |  |  | -0.025 | 0.024 | -0.023 | 0.025 |
| Summer × Lid on |  |  | -0.085 | 0.059 | -0.082 | 0.058 |
| Autumn × Without Lid on |  |  | -0.028 | 0.031 | -0.027 | 0.032 |
| Autumn × Lid on |  |  | -0.052^**^ | 0.019 | -0.056^**^ | 0.018 |
| Winter × Without Lid on |  |  | 0.008 | 0.038 | 0.010 | 0.039 |
| Winter × Lid on |  |  | -0.103^***^ | 0.012 | -0.097^***^ | 0.014 |
| *Season* × *Drinking alone* |  |  |  |  |  |  |
| Summer × Drinking with company |  |  | -0.035 | 0.021 | -0.034 | 0.021 |
| Summer × Drinking alone |  |  | -0.026 | 0.023 | -0.023 | 0.023 |
| Autumn × Drinking with company |  |  | -0.034 | 0.029 | -0.035 | 0.029 |
| Autumn × Drinking alone |  |  | -0.025 | 0.020 | -0.021 | 0.022 |
| Winter × Drinking with company |  |  | -0.014 | 0.034 | -0.013 | 0.034 |
| Winter × Drinking alone |  |  | 0.011 | 0.037 | 0.015 | 0.037 |
| Weekday × Added ingredients |  |  | -0.004^*^ | 0.002 | -0.004^*^ | 0.002 |
| Weekday × Black coffee |  |  | -0.001 | 0.004 | -0.002 | 0.004 |
| *Time of day* × *Cream* |  |  |  |  |  |  |
| Noon × No cream |  |  | -0.024^*^ | 0.011 | -0.024^*^ | 0.011 |
| Noon × Cream |  |  | 0.022 | 0.057 | 0.018 | 0.059 |
| Evening × No cream |  |  | 0.023 | 0.019 | 0.023 | 0.019 |
| Evening × Cream |  |  | 0.012 | 0.044 | 0.009 | 0.047 |
| *Time of day* × *Premium cup* |  |  |  |  |  |  |
| Noon × No premium cup |  |  | 0.017 | 0.019 | 0.014 | 0.018 |
| Noon × Premium cup |  |  | -0.031^*^ | 0.013 | -0.030^*^ | 0.013 |
| Evening × No premium cup |  |  | -0.016 | 0.037 | -0.012 | 0.039 |
| Evening × Premium cup |  |  | 0.032^*^ | 0.015 | 0.030^*^ | 0.015 |
| *Generation* × *Waking hour* |  |  |  |  |  |  |
| Boomers × Waking hour |  |  |  |  | -0.051^***^ | 0.016 |
| Gen X × Waking hour |  |  |  |  | -0.006 | 0.009 |
| Millennials × Waking hour |  |  |  |  | -0.009^*^ | 0.004 |
| Gen Z × Waking hour |  |  |  |  | 0.001 | 0.005 |
| *Generation* ×*Milk* |  |  |  |  |  |  |
| Gen X × Without Milk |  |  |  |  | 0.152^***^ | 0.045 |
| Gen X × Milk |  |  |  |  | 0.006 | 0.019 |
| Millennials × Without Milk |  |  |  |  | 0.142^***^ | 0.037 |
| Millennials × Milk |  |  |  |  | 0.025 | 0.028 |
| Gen Z × Without Milk |  |  |  |  | 0.148^***^ | 0.041 |
| Gen Z × Milk |  |  |  |  | 0.049 | 0.036 |
| *N* | 1,586 |  | 1,586 |  | 1,586 |  |

*Notes:* FR model with robust standard errors clustered by recruitment country (*n* = 8). These estimates are derived from the FR model in which the dependent variable, coffee Liking, was rescaled to the [0,1] interval. Thus, a margin measures the change in the expected proportion of the maximum score for a one-unit change in a covariate (with 1 as the highest possible liking on the original scale). The margins are computed by holding all other covariates in the model at their observed values. ^*^*p*<.05, ^**^*p*<.01, ^***^*p*<.001.

**Table S9.** Main effects across specifications for the main predictors.

|  | **Baseline FR^a^** | | **Temporal FR^a^** | | **Demographic FR^a^** | | **Baseline FR^b^** | | **Temporal FR^b^** | | **Demographic FR^b^** | |
| --- | --- | --- | --- | --- | --- | --- | --- | --- | --- | --- | --- | --- |
| Effect | *p* | *p-Holm* | *p* | *p-Holm* | *p* | *p-Holm* | *p* | *p-Holm* | *p* | *p-Holm* | *p* | *p-Holm* |
| Cream (joint) | .042 | .294 | .829 | 1 | .852 | 1 | .009 | .109 | .889 | 1 | .922 | 1 |
| Milk (joint) | .103 | .518 | .093 | .653 | .015 | .138 | .067 | .445 | .067 | .536 | <.001 | <.001 |
| Sugar (joint) | .007 | .066 | .006 | .076 | .004 | .045 | .005 | .065 | .008 | .083 | .002 | .022 |
| Black coffee (joint) | .296 | 1 | .026 | .211 | .030 | .185 | .424 | 1 | .095 | .574 | .097 | .489 |
| Lid on (joint) | .028 | .253 | .016 | .150 | .021 | .152 | .026 | .235 | .075 | .536 | .078 | .470 |
| Drinking alone (joint) | .029 | .253 | .453 | 1 | .419 | 1 | .124 | .497 | .399 | 1 | .324 | 1 |
| PPP-adj. price | <.001 | .004 | <.001 | .008 | <.001 | .007 | <.001 | <.001 | <.001 | <.001 | <.001 | <.001 |
| Premium cup (joint) | .045 | .294 | .013 | .135 | .013 | .131 | .063 | .445 | <.001 | <.001 | <.001 | <.001 |
| Number of coffee cups (joint) | <.001 | <.001 | <.001 | <.001 | <.001 | <.001 | <.001 | <.001 | <.001 | <.001 | <.001 | <.001 |
| Waking hour | .405 | 1 | .393 | 1 | .016 | .138 | .519 | 1 | .497 | 1 | <.001 | .002 |
| Season (joint) | <.001 | <.001 | <.001 | <.001 | <.001 | <.001 | .032 | .263 | .325 | 1 | .295 | 1 |
| Weekday (joint) | .370 | 1 | .299 | 1 | .287 | 1 | .565 | 1 | <.001 | <.001 | <.001 | <.001 |
| Time of the day (joint) | <.001 | <.001 | .857 | 1 | .814 | 1 | .022 | .227 | .756 | 1 | .699 | 1 |
| Generation (joint) | .285 | 1 | .275 | 1 | .167 | .839 | .076 | .445 | .052 | .474 | .037 | .263 |

*Notes:* ^a^FR model with robust standard errors; ^b^FR model with robust standard errors clustered by recruitment country. Reported statistics are joint Wald tests with robust variance/cluster-robust variance, where multi-parameter predictors (categorical factors and polynomial terms) are evaluated as blocks and continuous predictors as 1-df tests. Raw joint Wald *p*-value are shown alongside Holm–Bonferroni–adjusted *p*-values (p-Holm), computed within the focal predictor set to control the family-wise error rate at *α* = 0.05.

**Table S10.** Interaction effects across specifications. Benjamini–Hochberg FDR q-values within model.

|  | **Temporal FR^a^** | | **Demographic FR^a^** | | **Temporal FR^b^** | | **Demographic FR^b^** | |
| --- | --- | --- | --- | --- | --- | --- | --- | --- |
| Effect | p | q-BH | p | q-BH | p | q-BH | p | q-BH |
| Season×Lid on (joint) | <.001 | <.001 | <.001 | <.001 | <.001 | <.001 | <.001 | <.001 |
| Season×Drinking alone (joint) | <.001 | <.001 | <.001 | <.001 | <.001 | <.001 | <.001 | <.001 |
| Time of day×Cream (joint) | .064 | .080 | <.001 | <.001 | .065 | .092 | <.001 | <.001 |
| Time of day×Premium cup (joint) | <.001 | <.001 | <.001 | <.001 | <.001 | <.001 | <.001 | <.001 |
| Weekday×Black coffee (joint) | .104 | .104 | <.001 | <.001 | .128 | .128 | <.001 | <.001 |
| Generation×Waking hour (joint) |  |  |  |  | .025 | .044 | <.001 | <.001 |
| Generation×Milk (joint) |  |  |  |  | .116 | .128 | <.001 | <.001 |

*Notes:* ^a^FR model with robust standard errors are in parenthesis; ^b^FR model with robust standard errors clustered by recruitment country. Tests reported are joint Wald tests of each interaction block with robust variance. *p* means raw *p*-value from the joint test. *q*-BH means Benjamini–Hochberg *q*-value (FDR) computed within interaction variables set for each model.

**List of participating coffee venues^[[1]](#footnote-1),^^[[2]](#footnote-2),^^[[3]](#footnote-3)^**

**Extracted from the English language questionnaire**

*In Australia, in alphabetical order:* Brunswick Rex, Carte Crepes, Castro’s Kiosk, Disciple Coffee, Green Refectory, House of Cards, Loose Goose Café, Melbourne Connect, and The Good Bakers.

*In Norway:* Tim Wendelboe.

*In the UK, in alphabetical order:* Modern Coffee, Notes Coffee Roasters & Bar, Sky Garden, Story Coffee, Velasquez and Van Wezel, and Watch House.

*In the USA, in alphabetical order:* City Coffee Place NYC, Habitat Coffee Roasters, Panther Coffee, The Crown Oakland, and Watch House.

**Extracted from the Italian language questionnaire**

*In alphabetical order:* Bar Olimpia, Bowie Café, and Campus Café.

**Extracted from the Japanese language questionnaire**

*In alphabetical order:* Mermaid Coffee Roasters, Rec Coffee, and 2050 Coffee.

**Extracted from the Portuguese language questionnaire**

*In Brazil, in alphabetical order:* Ancora Coffee House, Casa do frade, Minas Gerais, Takko Café, and Zud Café.

**Extracted from the Romanian language questionnaire**

*In Romania, alphabetical order:* Acaju, August no2, Beans & Dots, Boiler Coffee Shop, Bujor, Cervo, Copac, Darlington, El Gato, Fabricuta de cafea, Fika, Frateria, Gist, Harmony, Kream, La Strada, Leche, Matala, Merlin, Meron Cluj, Noir, Prajitorul din Oz, Status, Two MiNotes Coffee shop, Teo’s Café, Tucano, Vitraj, Ziretto, 5 to Go, and 7am.

**Extracted from the Spanish language questionnaire**

*In Colombia, in alphabetical order:* Chapinero, COLO, Bacu, El Nogal, Engativa, Expedicion Café, Fontibon, Gran Estacion Bogota, Juan Valdez, Kennedy, La Tebaida, Masa, Punte Aranda, Quindio, Soprano, Storie d’amor, Tensaquillo, Tropicalia Coffee, Usaquen, Varietale

*In Spain, in alphabetical order:* Dr Fouquet, and Hola Coffee.

**Extracted from the Turkish language questionnaire**

*In Türkiye, in alphabetical order:* Adana Turkuaz Art, Artukbey, Asgard Coffee, Bielefeld, Deza Coffee, Dospresso, EspressoLab, Factory Istanbul, Finike Turucova, Ganos Coffee, Geek Coffee No 1, Geek Coffee No 2, Geek Coffee No 3, Geek Coffee No 4, Geek Coffee No 5, Gloria Jeans Coffee, HeffaLump, London Coffee, MackBear Coffee, Marin, Mikel Coffee, Pablo Artisan, Pause Coffee, Rio Coffee, Starbucks, Tekirdag Punch, Tuana Coffee, Tchibo, Yemen Coffee, and Zahara.

STROBE Statement—Checklist of items that should be included in reports of ***cross-sectional studies***

|  | Item No | Recommendation | Page No |
| --- | --- | --- | --- |
| **Title and abstract** | 1 | (*a*) Indicate the study’s design with a commonly used term in the title or the abstract | Title (p.1), Abstract & Keywords (p.2) |
|  |  | (*b*) Provide in the abstract an informative and balanced summary of what was done and what was found | Abstract (p.2) |
| Introduction | | |  |
| Background/rationale | 2 | Explain the scientific background and rationale for the investigation being reported | Introduction (pp.3-4) |
| Objectives | 3 | State specific objectives, including any prespecified hypotheses | Introduction (p.5) |
| Methods | | |  |
| Study design | 4 | Present key elements of study design early in the paper | Introduction (p.5) |
| Setting | 5 | Describe the setting, locations, and relevant dates, including periods of recruitment, exposure, follow-up, and data collection | Methods (Procedure) (p.20), Supp. Table S1 (p.1), and Supp. List of participating coffee venues (pp. 15-16) |
| Participants | 6 | (*a*) Give the eligibility criteria, and the sources and methods of selection of participants | Methods (Participants) (pp.18-19) and Methods (Procedure) (p.20) |
| Variables | 7 | Clearly define all outcomes, exposures, predictors, potential confounders, and effect modifiers. Give diagnostic criteria, if applicable | Methods (Apparatus and Materials) (pp. 19-20) |
| Data sources/ measurement | 8* | For each variable of interest, give sources of data and details of methods of assessment (measurement). Describe comparability of assessment methods if there is more than one group | Methods (Apparatus and Materials) (pp. 19-20) |
| Bias | 9 | Describe any efforts to address potential sources of bias | Methods (Participants & Procedure) (pp. 18-20) and Discussion (p.17-18) |
| Study size | 10 | Explain how the study size was arrived at | Methods (Participants) (pp.18-19) |
| Quantitative variables | 11 | Explain how quantitative variables were handled in the analyses. If applicable, describe which groupings were chosen and why | Results (Tables 1 & 2 and notes, p. 6), Information-theoretic data exploration (p.21) Complementary regression approaches to predict coffee Liking (p.23) |
| Statistical methods | 12 | (*a*) Describe all statistical methods, including those used to control for confounding | Methods (Data analysis, pp. 21-25; Welch’s ANOVAs , Information-theoretic exploration, Fractional and ZOIB regressions |
|  |  | (*b*) Describe any methods used to examine subgroups and interactions | Methods (Group-based data exploration, p.22); hierarchical specification for baseline, temporal, and demographic specifications (p. 22) |
|  |  | (*c*) Explain how missing data were addressed | N.A. |
|  |  | (*d*) If applicable, describe analytical methods taking account of sampling strategy | Robust standard errors clustered at recruitment country level (p.23) and Multilevel linear mixed model with random intercepts (p.24-25) |
|  |  | (*e*) Describe any sensitivity analyses | Multilevel linear mixed model with random intercepts (p.24-25) and Fractional regression without the Turkey subsample (p. 25) |
| Results | | |  |
| Participants | 13* | (a) Report numbers of individuals at each stage of study—eg numbers potentially eligible, examined for eligibility, confirmed eligible, included in the study, completing follow-up, and analysed | Results (pp.6-12) and all tables in the Supplementary file (pp.1-14) |
|  |  | (b) Give reasons for non-participation at each stage | Methods (Data Analysis/ Complementary regression approaches to predict coffee Liking, point 4, p.25, Turkish participants excluded from the analysis). |
|  |  | (c) Consider use of a flow diagram | N.A. |
| Descriptive data | 14* | (a) Give characteristics of study participants (eg demographic, clinical, social) and information on exposures and potential confounders | Results (p. 6, Table 1 & Table 2) |
|  |  | (b) Indicate number of participants with missing data for each variable of interest | Results (Sensitivity analysis in support of the coffee Liking prediction, pp.11-12) and Table S8. In Supplementary file (pp.12-13) |
| Outcome data | 15* | Report numbers of outcome events or summary measures | Results (Table 2 & Figure 1-3, p. 6); Figure S2 in Supplementary file (p.2) |
| Main results | 16 | (*a*) Give unadjusted estimates and, if applicable, confounder-adjusted estimates and their precision (eg, 95% confidence interval). Make clear which confounders were adjusted for and why they were included | Results (Fractional regression with robust/clustered standard errors in Figures 4&7, p. 7,12 and Supplementary Tables S3-S4, pp.5-9 and Supplementary Table S8, pp. 12-13, ZOIB model with robust standard errors in Figure 5, p. 9 and Supplementary Table S5, p.9, multilevel linear mixed regressions in Figure 6, p. 11 and Supplementary Tables S6-S7, pp.9-12). |
|  |  | (*b*) Report category boundaries when continuous variables were categorized | Results (Table 1 Notes, p. 6) and Information-theory exploratory results in Supplementary file (pp.3-5) |
|  |  | (*c*) If relevant, consider translating estimates of relative risk into absolute risk for a meaningful time period | N.A. |
| Other analyses | 17 | Report other analyses done—eg analyses of subgroups and interactions, and sensitivity analyses | Methods (Data Analysis, Complementary regression approaches to predict coffee Liking, pp. 22-25) and Results (Fractional response logit (FR) results to predict the overall coffee Liking score, Zero-One Inflated Beta (ZOIB) results for predicting perfect coffee Liking score, Robustness results to recruitment country geographical clustering, Sensitivity analysis in support of the coffee Liking prediction pp.7-12) with the Supplementary Tables S3-S8 (p.5-13) |
| Discussion | | |  |
| Key results | 18 | Summarise key results with reference to study objectives | Discussion (pp. 12-13) |
| Limitations | 19 | Discuss limitations of the study, taking into account sources of potential bias or imprecision. Discuss both direction and magnitude of any potential bias | Discussion (pp. 17-18) |
| Interpretation | 20 | Give a cautious overall interpretation of results considering objectives, limitations, multiplicity of analyses, results from similar studies, and other relevant evidence | Discussion (pp. 13-17) |
| Generalisability | 21 | Discuss the generalisability (external validity) of the study results | Discussion (pp. 18) |
| Other information | | |  |
| Funding | 22 | Give the source of funding and the role of the funders for the present study and, if applicable, for the original study on which the present article is based |  |

*Give information separately for exposed and unexposed groups.

**Note:** An Explanation and Elaboration article discusses each checklist item and gives methodological background and published examples of transparent reporting. The STROBE checklist is best used in conjunction with this article (freely available on the Web sites of PLoS Medicine at http://www.plosmedicine.org/, Annals of Internal Medicine at http://www.annals.org/, and Epidemiology at http://www.epidem.com/). Information on the STROBE Initiative is available at www.strobe-statement.org.

1. Most of the coffee shops listed below were contacted prior to data collection and acted as a host for our adverts for the present coffee study participation. However, adverts to participate in this coffee study were also strategically placed in several university campuses, therefore, we list here all coffee names submitted by our participants, even though the specific coffee shop may have not been contacted by our team for data collection. [↑](#footnote-ref-1)
2. Because important parts of the data were collected in coffee shops chains located in multiple cities of the participating countries, we provide only the name of the coffee shop/chain, and not the specific location. [↑](#footnote-ref-2)
3. 77 nationalities were reported (*N* = 2987), in descending absolute numbers: Turkey (1058), Romania (416), UK (395), Colombia (247), US (129), Japan (120), Australia (91), Brazil (86), Italy (62), Germany (30). And countries accounting for less than 1% of the sample (i.e., less than 29 participants): Ireland (27), Russia (20), France (20), Spain (19), Canada (19), China (17), India (13), Poland (11), Netherlands (10), New Zealand (9), Czech Republic (9), Malaysia (8), Belgium (8), Venezuela (7), Mexico (7), Israel (7), Cuba (7), Argentina (7), Singapore (6), Moldova (6), Greece (6), Taiwan (5), Sweden (5), Norway (5), Hungary (5), Hong Kong (5), Ukraine (4), Thailand (4), Switzerland (4), Philippines (4), South Korea (4), Denmark (4), Austria (4), South Africa (3), Slovakia (3), Saudi Arabia (3), Kuwait (3), Uruguay (2), Qatar (2), Nigeria (2), Finland (2), Dominican Republic (2), Cyprus (2), Belarus (2), UAE (1), Somalia (1), Portugal (1), Peru (1), Macedonia (1), Lithuania (1), Lybia (1), Kenya (1), Iran (1), Indonesia (1), Guatemala (1), Grenada (1), Ghana (1), Ethiopia (1), Estonia (1), El Salvador (1), Egypt (1), Ecuador (1), Croatia (1), Bulgaria (1), Bolivia (1), Bangladesh (1), Bahamas (1), no nationality provided (8). [↑](#footnote-ref-3)
